# PARD3 drives tumorigenesis through activating Sonic Hedgehog signalling in tumour-initiating cells in liver cancer

**DOI:** 10.1186/s13046-024-02967-3

**Published:** 2024-02-06

**Authors:** Junyu Wu, Hor-Yue Tan, Yau-Tuen Chan, Yuanjun Lu, Zixin Feng, Hongchao Yuan, Cheng Zhang, Yibin Feng, Ning Wang

**Affiliations:** 1 School of Chinese Medicine, LKS Faculty of Medicine, The University of Hong Kong, Hong Kong SAR, China; 2 Centre for Chinese Medicine Drug Development, School of Chinese Medicine, Hong Kong Baptist University, Hong Kong SAR, China

**Keywords:** PARD3, Hepatic tumorigenesis, Cancer stem cells, Sonic hedgehog, Berberine

## Abstract

**Background:**

Par-3 Family Cell Polarity Regulator (PARD3) is a cellular protein essential for asymmetric cell division and polarized growth. This study aimed to study the role of PARD3 in hepatic tumorigenesis.

**Methods:**

The essential role of PARD3 in mediating hepatic tumorigenesis was assessed in diet-induced spontaneous liver tumour and syngeneic tumour models. The mechanism of PARD3 was delineated by bulk and single-cell RNA sequencing. The clinical significance of PARD3 was identified by tissue array analysis.

**Results:**

PARD3 was overexpressed in tumour tissues and PARD3 overexpression was positively correlated with high tumour stage as well as the poor prognosis in patients. In models of spontaneous liver cancer induced by choline-deficient, amino acid-defined (CDAA) and methionine-choline-deficient (MCD) diets, upregulation of PARD3 was induced specifically at the tumorigenesis stage rather than other early stages of liver disease progression. Site-directed knockout of PARD3 using an adeno-associated virus 8 (AAV8)-delivered CRISPR/Cas9 single-guide RNA (sgRNA) plasmid blocked hepatic tumorigenesis, while PARD3 overexpression accelerated liver tumour progression. In particular, single-cell sequencing analysis suggested that PARD3 was enriched in primitive tumour cells and its overexpression enhanced tumour-initiating cell (TICs). Overexpression of PARD3 maintained the self-renewal ability of the CD133^+^ TIC population within hepatocellular carcinoma (HCC) cells and promoted the in vitro and in vivo tumorigenicity of CD133^+^ TICs. Transcriptome analysis revealed that Sonic Hedgehog (SHH) signalling was activated in PARD3-overexpressing CD133^+^ TICs. Mechanistically, PARD3 interacted with aPKC to further activate SHH signalling and downstream stemness-related genes. Suppression of SHH signalling and aPKC expression attenuated the in vitro and in vivo tumorigenicity of PARD3-overexpressing CD133^+^ TICs. Tissue array analysis revealed that PARD3 expression was positively associated with the phosphorylation of aPKC, SOX2 and Gli1 and that the combination of these markers could be used to stratify HCC patients into two clusters with different clinicopathological characteristics and overall survival prognoses. The natural compound berberine was selected as a potent suppressor of PARD3 expression and could be used as a preventive agent for liver cancer that completely blocks diet-induced hepatic tumorigenesis in a PARD3-dependent manner.

**Conclusion:**

This study revealed PARD3 as a potential preventive target of liver tumorigenesis via TIC regulation.

**Supplementary Information:**

The online version contains supplementary material available at 10.1186/s13046-024-02967-3.

## Backgrounds

Primary liver cancer is the sixth most common cancer and the third deadliest cancer worldwide, with 41,210 new cases and 29,380 deaths estimated to occur in 2023 in the USA alone [1, 2]. Hepatocellular carcinoma (HCC) is the predominant primary liver cancer and is usually diagnosed at a late stage with a dismal prognosis. In general, HCC tends to arise in individuals who are genetically predisposed and possess one or more risk factors, leading to the gradual establishment of a conducive milieu for the initiation and progression of carcinogenesis [3]. The risk factors of HCC are quite complex. Previously, hepatitis virus infection was an important cause of HCC, accounting for nearly one-quarter of HCC cases. However, with effective interventions with antiviral drugs and vaccines, the proportion of hepatitis-related HCC cases has gradually decreased, while the proportion of HCC cases caused by metabolic liver disease have gradually increased due to the recent prevalence of metabolic diseases such as obesity [4]. Indeed, non-alcoholic fatty liver disease (NAFLD), which is driven by components of metabolic syndrome such as obesity, was confirmed to contribute exponentially to the incidence of HCC and has become the most rapidly increasing cause of HCC [4–6]. Due to the lack of effective early screening approaches, patients with NAFLD-driven HCC are diagnosed at relatively late stages and with substantial involvement, because NAFLD-associated HCC tends to develop in the absence of liver cirrhosis [7, 8]. The abysmal surveillance substantially worsens the prognosis of NAFLD-associated HCC. Therefore, deciphering the molecular mechanisms of hepatocarcinogenesis and discovering early biomarkers are clinically imperative needs.

HCC is considered a heterogeneous disease in which the tumour bulk harbours a pool of cancer cells with diverse genetic expression signatures and molecular functions. The heterogeneity of HCC is maintained by a subpopulation of cells with stem cell-like properties, namely, tumour-initiating cells (TICs). TICs give rise to the heterogeneous structure of tumours and account for the therapeutic failure and recurrence of HCC due to their self-renewal and differentiation abilities [9]. The molecular mechanisms involved in early hepatocellular carcinogenesis remain to be elucidated. However, existing evidence suggests that TICs play a fundamental role in hepatocarcinogenesis [10, 11]. Specifically, liver progenitor cells, the cellular source of carcinogenesis, undergo a series of molecular events caused by chronic liver diseases, including but not limited to sustained DNA damage and increased chromosomal instability [12, 13]. Thus, liver progenitor cells gradually transform into TICs, which additionally initiate tumorigenesis and progression [14]. The signalling pathways and molecules that maintain the stemness of TICs have been a popular topic in cancer treatment research. Par-3 family cell polarity regulator (PARD3) is a member of the Par polarity complex, which governs cell polarity [15]. PARD3 plays important roles in biological processes related to spatial asymmetry, such as asymmetrical cell division (ACD), which is notably highly conserved in TICs. By ACD, TICs give rise to a bulk tumour cell population while maintaining a daughter cell population with stemness properties [16]. PARD3 is reported to be dysregulated and to participate in the progression of several types of malignancies [17–20]. Recent studies have suggested that the cell polarity machinery is potentially involved in oncogenic activities during the cell transformation process [21, 22]. However, the specific mechanism by which PARD3 regulates hepatocarcinogenesis remains unclear.

In this study, we systematically investigated the role of PARD3 in hepatocarcinogenesis. We discovered that PARD3 was upregulated in the tumorigenesis stage in a mouse model of spontaneous HCC. The essential role of PARD3 in diet-induced hepatocarcinogenesis was verified by both loss- and gain-of-function studies. PARD3 overexpression resulted in enrichment of CD133^+^ TICs in an orthotopic HCC model. Mechanistically, PARD3 maintains the stemness of CD133^+^ cells by activating sonic hedgehog (SHH) signalling. We demonstrated that PARD3 is a potential target for the inhibition of hepatocarcinogenesis.

## Methods

### Animal experiments

Protocols of all the animal studies were reviewed and approved by the Committee of Use of Laboratory Animal in Teaching and Research (CULATR) in the University of Hong Kong.

Study 1. Four-week-old C57BL/6J mice were fed a choline-deficient, amino acid-defined (CDAA) diet or Methionine and Choline-Deficient (MCD) diet (Dyets Inc., PA, USA) for an indicated period. To examine the effects of PARD3 knockout on carcinogenesis in vivo, mice were subjected to intravenous injection of either (i) adeno-associated virus 8 (AAV8)-vector or (ii) AAV8-PARD3KO (1 × 10^11^ viral genomes/mouse) (GenePharma, China) every two weeks (*n* = 10). To examine the effects of PARD3 overexpression on carcinogenesis in vivo, mice were and subjected to intravenous injection of either (i) AAV8-vector or (ii) AAV8-PARD3Act (1 × 10^11^ viral genomes/mouse) (GenePharma, P.R. China) every two weeks (*n* = 10). For berberine intervention, mice were subjected to intraperitoneal injection of berberine (10 mg/kg/3 days, i.p.) for an indicated period. At the end of the experiment, the liver tissues were harvested and examined for any morphological changes.

Study 2. The role of PARD3 in liver cancer progression was evaluated in an orthotopic liver cancer mouse model. Specifically, 2 × 10^6^ Hepa1-6 cells stably transfected with the PARD3 or empty plasmid were suspended in 30 µl of serum-free DMEM/Matrigel (1:1) and injected into the left liver lobe of 5-week-old C57BL/6J mice. Three weeks after tumour cell implantation, the liver tumours were harvested and weighed.

Study 3. To determine the cancer-initiating cell frequency among CD133 + and CD133- PARD3-overexpressing (OE) cells, an in vivo limiting dilution assay was performed on cells isolated from NOD.CB17-Prkdc^scid^/J mice. Serial dilutions of cell suspensions (from 10^2^ to 10^4^) were subcutaneously injected into NOD.CB17-Prkdc^scid^/J mice. Tumour formation was continuously monitored for 3–8 weeks after subcutaneous injection.

### Single-cell RNA sequencing (scRNA-seq) and data analysis

Early-stage orthotopic tumours derived from PARD3-overexpressing and wild-type (WT) Hepa1-6 cells were excised from C57BL/6J mice 10 days after tumour cell implantation. Tumour tissues were digested in 8 mg/10 ml collagenase IV in PBS at 37 °C for 30 min. The undigested pellets were discarded, and cells in the supernatant were collected by centrifugation at 400 × g for 5 min. The cell pellets were resuspended in PBS containing 0.5% BSA to obtain single-cell suspensions before single-cell encapsulation and library preparation, which was performed at the Centre for PanorOmic Sciences, The University of Hong Kong. Uniquely individually barcoded RNA was obtained by using the Chromium Single Cell 3′ Library, Gel Bead & Multiplex Kit, and Chromium Single Cell Chip Kit following the instructions provided by 10x Genomics. RNA-seq was performed on the NovaSeq 6000 platform. Chromium single-cell data were processed with Cell Ranger software (v 6.1.2, 10x Genomics) to align reads and generate feature-barcode matrices. The Seurat object was constructed based on the gene expression matrix and metadata using the Seurat R package (v 4.0). In the quality control (QC) process, single cells with read counts > 5000 or < 200 and > 20% mitochondrial read counts were excluded from subsequent downstream analyses.

### Multiplex immunohistochemistry (IHC)

Multiplex IHC was conducted on a tissue microarray composed of 90 human HCC samples from patients with corresponding clinicopathological information purchased from Shanghai OUTDO Biotech Co., Ltd, Shanghai, China (LivH180Su17). Multiplex IHC was performed following the instructions provided by Akoya Biosciences (NEL797B001KT, PerkinElmer, Massachusetts, USA). In brief, the tissue slides were dewaxed, rehydrated and fixed before being subjected to epitope retrieval. Endogenous peroxidase was blocked with Antibody Diluent/Block reagent. Then, the slides were incubated with a primary antibody overnight prior to horseradish peroxidase (HRP) labelling and Opal signal generation. Before staining of the next target protein, the primary antibody-secondary antibody-HRP complexes formed in the current step were eluted by boiling in AR6 buffer. The primary antibodies against SOX2 (A0561, ABclonal), Gli1 (V812, Cell Signaling Technology), phospho-aPKC (9378, Cell Signaling Technology), PARD3 (11085-1-AP, Proteintech), and CD133 (A0818, ABclonal) were labelled with Opal 480, Opal 520, Opal 570, Opal 650 and Opal 780, respectively. After staining of all targets, the slide was counterstained with DAPI and imaged on a Vectra Polaris system (PerkinElmer, Massachusetts, USA). Further data analysis was carried out using inForm Advanced Image Analysis software (inForm 2.4.0; PerkinElmer, Massachusetts, USA). The 6 multispectral fluorescence signals were unmixed using a built-in library within inForm. Adaptive cell segmentation was performed to segment the nucleus, cytoplasm and membrane of cells in each tissue.

### Statistical analysis

All statistical analyses were conducted using GraphPad Prism 8 software (CA, USA). For data with a normal distribution, we used two-tailed unpaired Student’s t test for two‐group comparisons. For data with a non‐normal distribution, we used the Mann‒Whitney U test for two‐group comparisons. All data are presented as the means ± SDs. A *P* value of less than 0.05 was considered to indicate a statistically significant difference.

## Results

### PARD3 was upregulated during hepatic tumorigenesis

To explore the role of PARD3 in mediating hepatic tumorigenesis, we first extracted human data from the TCGA and GEO databases. PARD3 expression was significantly upregulated in tumour tissues compared with adjacent normal tissues of patients with HCC (Fig. S1A). PARD expression was positively correlated with TNM stage (Fig. S1B). High expression of PARD3 in tumour tissues was correlated with poor overall survival and recurrence-free survival in patients with liver cancer (Fig. S1C). As liver cancer is a long-term, progressively developing disease, we then examined whether PARD3 expression correlates with the progression of liver diseases towards liver cancer. We extracted human data from GEO dataset GSE25097 and found that interestingly, PARD3 expression was not significantly induced at the stage of liver cirrhosis but was sharply induced at the stage of hepatic tumorigenesis (Fig. S1D). As hepatic carcinogenesis can result from either viral infection- or metabolic-associated liver inflammation, we stratified patients in the TGCA database based on their medical history of viral infection. Interestingly, in liver cancer patients with a medical history of viral infection, the prognosis was not found to be correlated with the expression of PARD3. However, in patients without a history of hepatic viral infection, high expression of PARD3 was associated with unfavorable overall and recurrence-free survival. (Fig. S1E).

**Fig. 1:**
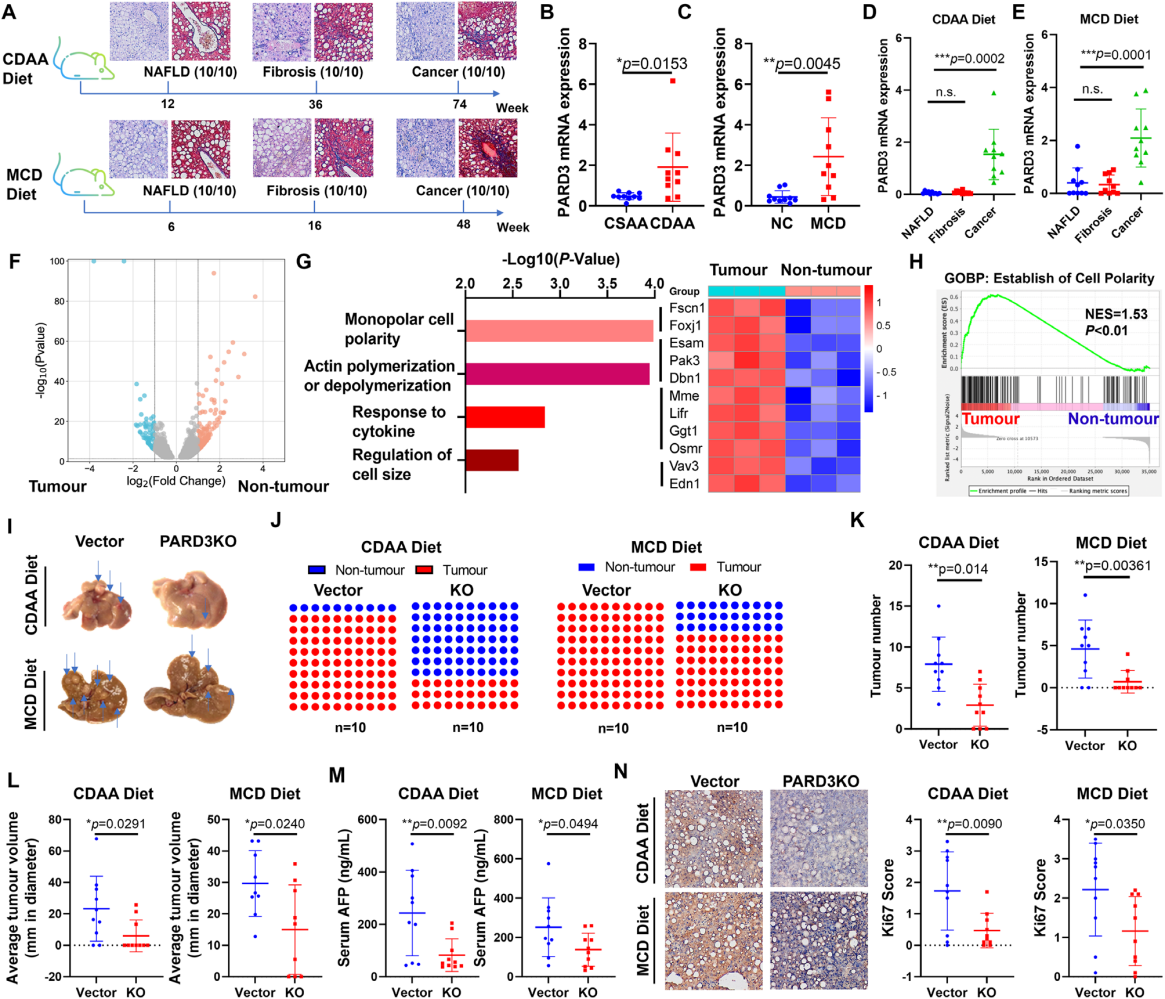
Expression profile of PARD3 during metabolic dysfunction-associated liver carcinogenesis. **A.** Diagram showing the process for establishment of the CDAA diet- and MCD diet-induced mouse hepatocarcinogenesis models. **B&C.** The relative mRNA expression level of PARD3 in mice with diet-induced liver cancer and their control diet-fed littermates. **D&E.** Expression of PARD3 was induced in the tumorigenesis stage rather than in metabolic liver disease stages. **F. **Transcriptional analysis of liver with or without surface tumours from C57BL/J mice after CDAA diet feeding for 54 weeks.** G.** GO enrichment analysis and heatmap of upregulated polarity-related genes in livers with tumour nodules compared with tumour-free livers. **H.** GSEA results showing that the establishment of cell polarity pathway was enriched in CDAA diet-fed mice with tumour nodule development. Upon PARD3 knockout, the hepatic **I.** tumour burden, **J.** tumour incidence, **K.** tumour number and **L.** tumour volume were significantly reduced in CDAA diet- and MCD diet-fed mice. PARD3 knockout significantly reduced the expression of **M.** AFP and decreased the **N.** Ki67^+^ cell population. *P < 0.05; **P < 0.01; ***P < 0.001; n.s., not statistically significant

We therefore examined the expression of PARD3 in the liver during metabolic dysfunction-associated liver carcinogenesis. To this end, we established models of CDAA diet-induced and methionine-choline-deficient (MCD) diet-induced liver carcinogenesis in C57BL/J mice (Fig. 1A). The livers were collected after different numbers of weeks of diet feeding to represent different stages of liver diseases, including fatty liver, fibrosis and cancer. Consistent with our analysis of publicly available human HCC data, the expression of PARD3 in mice with liver cancer was significantly higher than that in littermate mice fed control diets (Fig. 1B&C). Expression of PARD3 was not induced at earlier stages of liver diseases but was specifically upregulated at the tumorigenesis stage (Fig. 1D&E). To confirm the involvement of the PARD3-related pathway in hepatic carcinogenesis, we extracted total RNA from livers with or without surface tumours from C57BL/J mice after CDAA diet feeding for 54 weeks for transcriptomic analysis (Fig. 1F). Genes related to the establishment of cell polarity were enriched in the livers with tumours, as revealed by Gene Ontology (GO) enrichment analysis and gene set enrichment analysis (GSEA) (Fig. 1G&H). This observation suggested that PARD3 expression was specifically induced at the tumorigenesis stage of long-term liver disease.

### PARD3 knockout suppressed hepatic tumorigenesis

To examine whether PARD3 expression is essential for metabolic stress-induced hepatic carcinogenesis, we used AAV-mediated gene transfer to conditionally knock out the hepatic expression of PARD3 in the murine models of CDAA diet-induced and MCD diet-induced liver cancer (Fig. S2A). AAV8-mediated delivery of a CRISPR/Cas9 single-guide RNA (sgRNA) against PARD3 specifically silenced the expression of PARD3 in the mouse liver, as confirmed by immunohistochemical staining (Fig. S2B). No large differences in body weight changes were observed between mice with and mice without hepatic knockout of PARD3 (Fig. S2C). Serum ALT and AST levels were minimally changed upon hepatic knockout of PARD3 (Fig. S2D). H&E staining revealed that liver inflammation was not affected by PARD3 knockout, and Masson trichrome (MT) staining showed no large difference in hepatic fibrosis between mice with PARD3 knockout and their wild-type littermates (Fig. S2E). However, as expected, PARD3 knockout significantly reduced the tumour burden in both CDAA diet-fed and MCD diet-fed mice (Fig. 1I). The incidence of tumours on the livers of mice was decreased upon PARD3 knockout (Fig. 1J), and both the number and volume of tumours were reduced (Fig. 1K&L). PARD3 knockout significantly reduced the expression of the tumour marker AFP in the liver (Fig. 1M), as well as the population of Ki67^+^ cells (Fig. 1N). These findings suggest that PARD3 is essential for driving hepatic tumorigenesis in mice exposed to metabolic stress.

### PARD3 overexpression accelerated liver cancer progression

**Fig. 2:**
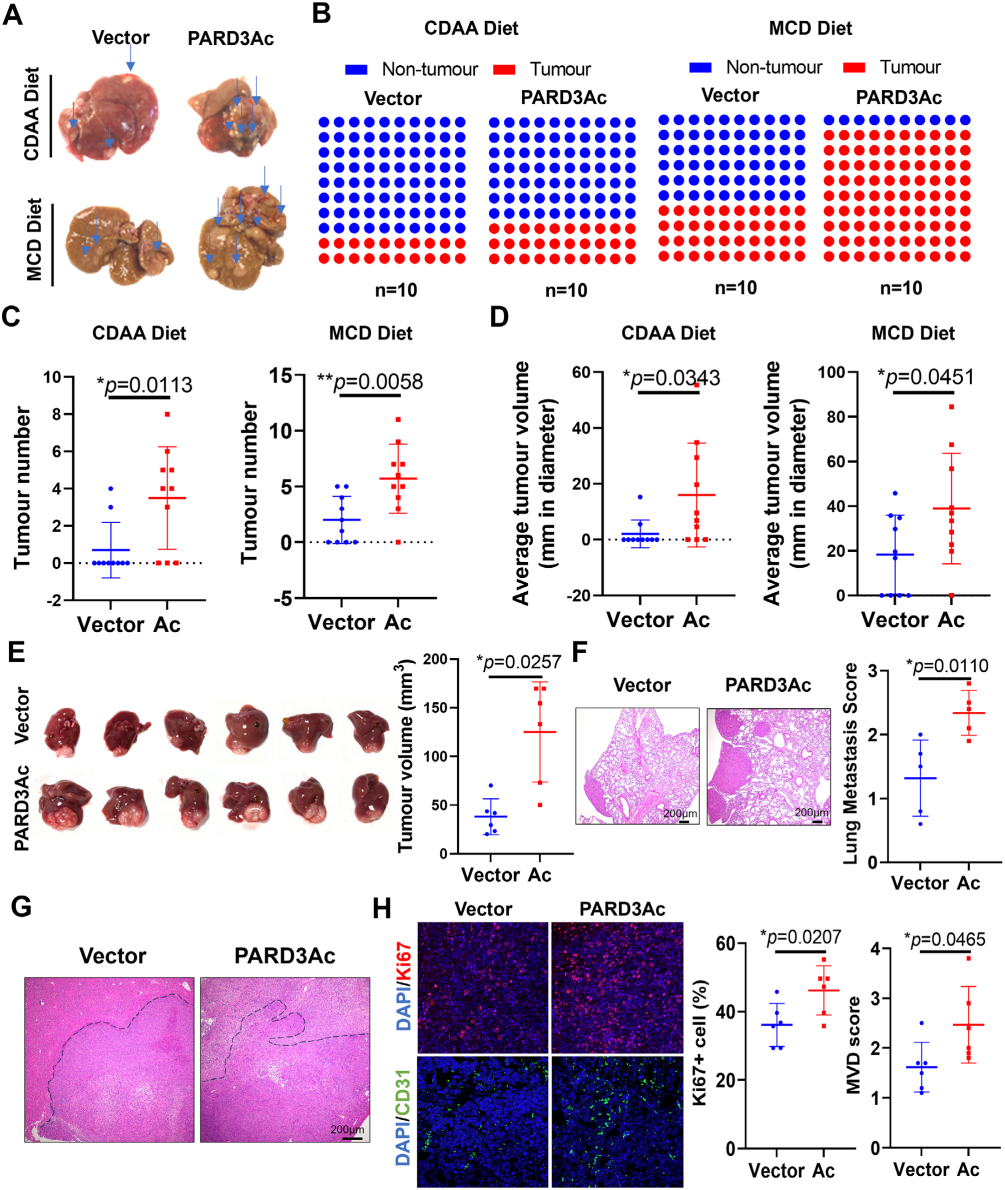
PARD3 overexpression accelerated liver cancer progression. PARD3 overexpression resulted in significantly higher hepatic **(A)** tumour burden, **(B)** tumour incidence, **(C)** tumour multiplicity and **(D)** tumour volume in CDAA diet- and MCD diet-fed mice. **(E)** Tumours derived from PARD3-overexpressing Hepa1-6 cells exhibited accelerated progression in the orthotopic liver cancer model. **(F)** PARD3 overexpression resulted in increased lung metastasis. **(G)** H&E staining on the liver tumour sections revealed a more pronounced invasiveness at the tumour periphery in PARD3 overexpression orthotopic HCC. **(H)** Higher CD31 and Ki67 expression was observed in PARD3-overexpressing Hepa1-6 orthotopic tumour tissue sections. *P < 0.05; **P < 0.01; ***P < 0.001; n.s., not statistically significant

To further analyse the role of PARD3 in mediating early-stage hepatic carcinogenesis, we specifically overexpressed PARD3 in the livers of C57BL/J mice via intraperitoneal injection of AAV8 for CRISPR activation of PARD3 (Fig. S3A&S3B). Mice with and without PARD3 overexpression were fed a CDAA diet for 54 weeks or an MCD diet for 32 weeks for investigation of whether PARD3 overexpression spontaneously induces and accelerates the formation of liver tumours. No large difference in body weight was observed. Serum ALT activity and serum AST activity were observed in mice with PARD3 overexpression compared with their wild-type littermates (Fig. S3C&S3D). Examination of livers revealed that only 20% of the wild-type mice fed the CDAA diet developed visible tumours, while mice fed the MCD diet developed more hepatic tumours, suggesting that the MCD diet is a stronger inducer of metabolic stress than the CDAA diet. Overexpression of PARD3 induced the development of more hepatic tumours in mice fed the 54-week CDAA and 32-week MCD diet (Fig. 2A&B) and resulted in increased tumour multiplicity and volume (Fig. 2C&D).

To confirm the role of PARD3 in promoting the progression of established liver cancer, we established a stable clone of PARD3-overexpressing Hepa1-6 cells, a murine liver cancer cell line isolated from C57BL/J mice (Fig. S3E). PARD3 overexpression in Hepa1-6 cells significantly induced cell proliferation, migration and invasion in vitro (Fig. S3F-H). We then injected 2 × 10^6^ Hepa1-6 cells with or without PARD3 overexpression into the left liver lobe of C57BL/J mice. PARD3 overexpression significantly accelerated in vivo tumour growth in the liver (Fig. 2E) and increased the incidence of lung metastasis of liver tumour cells (Fig. 2F). H&E staining on the liver tumour sections revealed a more pronounced invasiveness at the tumour periphery in PARD3 overexpression orthotopic HCC mice (Fig. 2G). Immunohistochemical analysis suggested that CD31 and Ki67 expression in PARD3-overexpressing Hepa1-6 tumours was higher than that in wild-type Hepa1-6 tumours (Fig. 2H). These observations suggest that PARD3 not only drives oncogenesis but also promotes tumour progression in the liver.

### Effects of PARD3 overexpression in the early tumour ecosystem at single-cell resolution

**Fig. 3:**
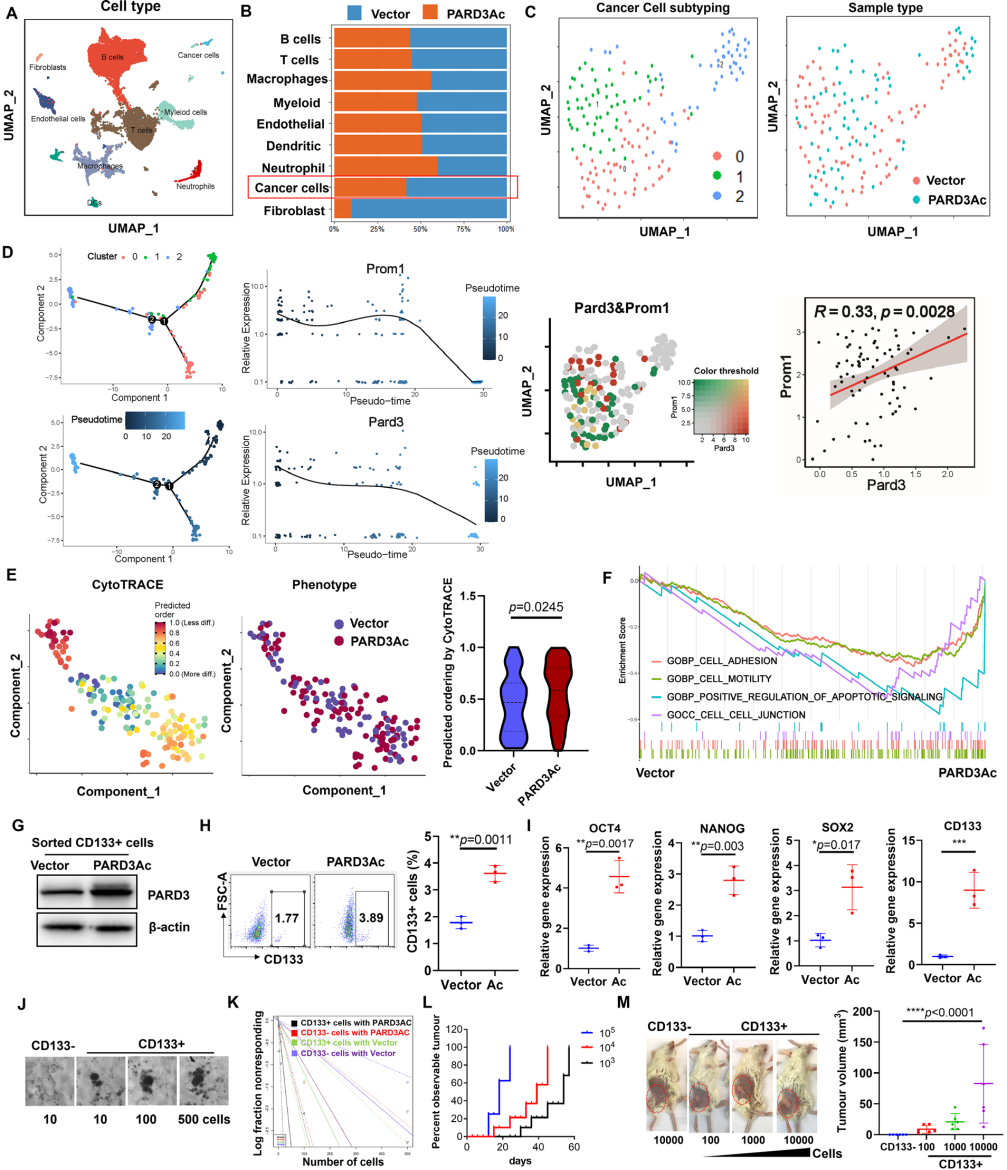
Single-cell sequencing analysis revealed that PARD3 maintained stemness of TICs in the early tumour ecosystems of HCC. **(A)** Uniform manifold projection and approximation (UMAP) plot showing the main cell lineages in early tumour ecosystems. **(B)** Proportions of various cell lineages identified in PARD3-overexpressing and wild-type early tumour tissues. **(C)** Cancer cells were additionally classified into 3 subclusters. **(D)** Trajectories of the 3 subclusters of cancer cells. Left panel: The expression patterns of PARD3 and Prom1 along the phylogenetic tree are visualized. Right panel: Pard3 and Prom1 are positively correlated in cluster 0 and 1 with lower differentiation potency. **(E)** CytoTRACE analysis of cancer cells. The CytoTRACE scores are visualized in a violin plot. **(F)** GSEA revealed the activated pathway in PARD3-overexpressing tumour cells in the early tumour ecosystem. **(G)** Immunoblotting showing the overexpression of PARD3 in CD133^+^ Hepa1-6 cells. **H&I.** PARD3 overexpression increased the CD133^+^ cell percentage and stemness-associated gene expression in Hepa1-6 cells. **J&K.** PARD3-overexpressing CD133^+^ Hepa1-6 cells showed stronger tumour sphere formation ability than their wild-type counterpart, as measured by an in vitro limiting dilution assay. **L&M.** An in vivo limiting dilution assay showed that the tumorigenic ability of CD133 + Hepa1-6 cells was enhanced by PARD3 overexpression. *P < 0.05; **P < 0.01; ***P < 0.001; n.s., not statistically significant

To comprehensively investigate the effects of PARD3 overexpression on the developmental and functional dynamics of early tumour ecosystems, we collected 21,294 cells from early-stage orthotopic murine liver tumours derived from wild-type and PARD3-overexpressing Hepa1-6 cells and subjected these cells to scRNA-seq. We identified 15 distinctive cell clusters containing immune, stromal and cancer cells. The 5 most specific cell markers of each cluster are shown in Fig. S4A. As shown in Fig. 3A&S4B, most cell lineages in early-stage orthotopic murine tumours were composed of adaptive immune cells, including B cells (Cd79a, Cd79b) and T cells (Cd3g, Cd3e). The innate immune cells comprised macrophages (Csf1r, Cd14), dendritic cells (Flt3, Xcr1), neutrophils (Cxcr2, Ccr1) and other myeloid cells (Tcf7, Il7r). The stromal cells comprised mainly fibroblasts (Col1a2, Col3a1) and endothelial cells (Flt1, Col4a1). Malignant cancer cells were defined by high expression of Afp, Sox9 and Epcam. PARD3 wild-type and overexpressing cells were not distinguishable by the composition of major cell lineages except for fibroblasts (Fig. 3B), which were clearly more prevalent in the PARD3-overexpressing tumour microenvironment. To delineate the transcriptional dynamics and heterogeneity of these cancer cells, we additionally clustered them into 3 subgroups, which were relatively evenly distributed between the wild-type and overexpressing groups (Fig. 3C). Pseudotime analysis was performed to map the pseudotemporal ordering of cell lineages, in which the 3 subgroups of cancer cells had different developmental statuses (Fig. 3D). Specifically, the majority of the cells in clusters 0 and 1 were distributed at the root of the phylogenetic tree, suggesting that they were likely to be primitive cancer cells. However, the cells in cluster 2 were located mainly at the extremities of the phylogenetic tree, suggesting a more differentiated state. Notably, both PARD3 and Prom1 (encoding CD133) showed preferential expression in cancer cells at early developmental stages, highlighting their potential role in maintaining the stem-like properties of malignancy (Fig. 3D). Intriguingly, both PARD3 and Prom1 were preferentially enriched in cancer cells of subgroups 0 and 1 and were positively correlated, suggesting that PARD3 and Prom1 share a similar expression pattern. To better describe the stemness of cancer cells, we further adopted the CytoTRACE algorithm, which allows a more precise evaluation of cancer stemness by measuring the differentiation potential. As shown in Fig. 3E, PARD3 overexpression resulted in a significantly higher CytoTRACE score, which indicated enhanced stemness. GSEA of the differentially expressed genes between PARD3 wild-type and overexpressing cancer cells showed enrichment of these genes in gene sets related to cell motility, cell adhesion and epithelial-to-mesenchymal transition (EMT) in PARD3-overexpressing cells (Fig. 3F), further indicating that EMT and stemness are positively correlated in cancer. To evaluate the dynamic microenvironmental changes in early-stage tumours between the PARD3 wild-type and overexpressing groups, we analysed cell‒cell communication strength with CellChat (Fig. S4C). Communication between cancer cells and T cells was attenuated, suggesting that the increased stemness potential conferred by PARD3 overexpression may endow a higher potential for immune evasion. In contrast, cancer cell–cancer cell, cancer cell–fibroblast and cancer cell–myeloid cell communication was apparently upregulated in the PARD3 overexpressing early-stage tumour microenvironment. Correspondingly, we conducted GSEA on fibroblasts and myeloid cells to explore the potential functional changes induced by PARD3 overexpression in cancer cells (Fig. S4D). Lipid metabolism was activated in myeloid cells of PARD3 overexpressing tumours. In fibroblasts, immune cell chemotaxis and leukocyte migration were suppressed, suggesting that the chemoattractant ability of fibroblasts was weakened by PARD3 overexpression. These observations at single-cell resolution showed that a heterogeneous tumour population formed at the early stage of orthotopic tumorigenesis in mice, with PARD3 playing an important role in maintaining the stemness of cancer cells. In addition, PARD3 overexpression potentially affected the ecosystem of early-stage tumours, as shown by the abundant fibroblast population and attenuated interactions with T cells.

### PARD3A maintained the self-renewal of CD133+ TICs

To further explore the role of PARD3 as an oncogenic driver of hepatic tumorigenesis, we analysed the CD133^+^ cell population within Hepa1-6 cells with and without PARD3 overexpression using flow cytometry and found that PARD3 overexpression induced an increase in the CD133^+^ cell percentage among Hepa1-6 cells (Fig. 3G and H) and induced the expression of stemness-associated genes in CD133 + cells (Fig. 3I). To confirm that the PARD3 overexpression-induced CD133^+^ cell population acquired tumour-initiating properties, we used an in vitro limiting dilution assay to measure the sphere-forming ability of PARD3-overexpressing CD133^+^ Hepa1-6 cells. CD133^+^ cells isolated from PARD3-overexpressing cells showed the ability to form spheres in a cell density-dependent manner, indicating their tumorigenic properties (Fig. 3J&K). We then transplanted the CD133^+^ population isolated from PARD3-overexpressing Hepa1-6 cells into NOD.CB17-Prkdc^scid^/J mice to perform the in vivo limiting dilution assay. The CD133^+^ population formed tumours in vivo in a cell density-dependent manner (Fig. 3L), while the CD133^−^ population could not form tumours in vivo ​even at 10000 cells (Fig. 3M). These observations suggested that PARD3 overexpression led to a CD133^+^ cell population with self-renewal ability that exhibited tumour-initiating properties and was able to form tumours in vivo.

**Fig. 4:**
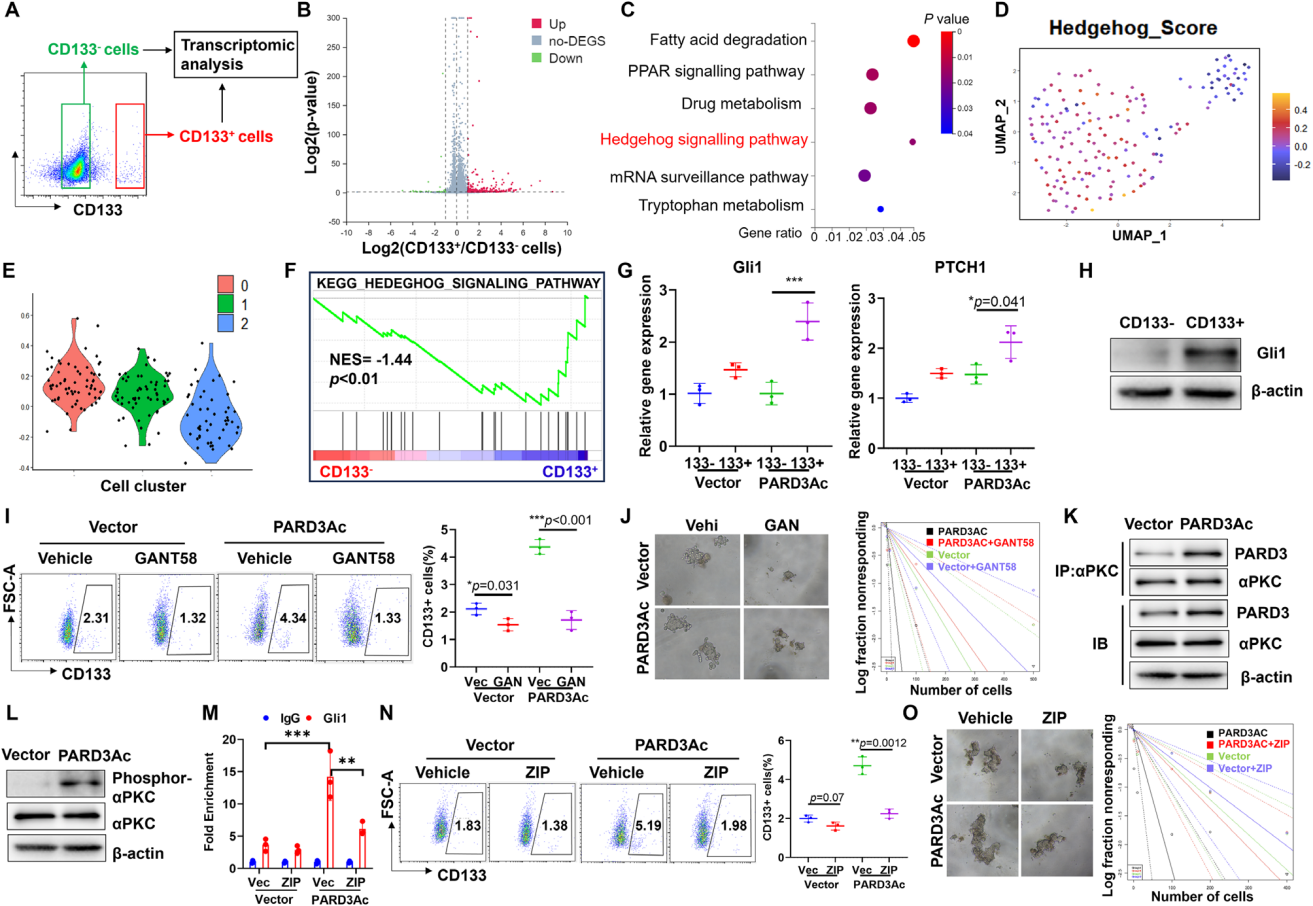
SHH signalling activation was responsible for stemness maintenance in PARD3-overexpressing CD133^+^ TICs. **(A)** CD133^+^ and CD133^−^ cells were sorted from PARD3-overexpressing Hepa1-6 cells and subjected to RNA-seq analysis. **(B)** PARD3 activation induced perturbation of gene expression in CD133^+^ cells compared with CD133^−^ cells. **(C)** Significant signalling pathways activated in CD133^+^ PARD3-overexpressing cells, as revealed by functional enrichment analysis. **D&E.** SHH signalling activity in different subclusters of cancer cells, as determined by single-cell sequencing. **F.** GSEA results showing that SHH signalling was activated in CD133^+^ PARD3-overexpressing cells. **G.** Relative expression of Gli1, PTCH1 and SHH in CD133^+^ and CD133^−^ PARD3-overexpressing cells. **H.** Gli1 protein expression was increased in CD133^+^ PARD3-overexpressing cells. **I.** Quantification of the CD133^+^ population within PARD3-overexpressing and wild-type Hepa1-6 cells treated with GANT58 (*n* = 3). **J.** In vitro limiting dilution assay showing the tumour sphere formation ability of PARD3 OE/wild-type CD133^+^ cells with or without GANT58 treatment. **K.** The interaction between aPKC and PARD3 was increased upon PARD3 overexpression, as determined by co-IP. **L.** The phosphorylation of aPKC was increased upon PARD3 overexpression. **M.** Gli1 binding to the Sox2 promoter was attenuated by ZIP. **N.** Quantification of the CD133^+^ population within PARD3-overexpressing Hepa1-6 cells with or without inhibition of aPKC signalling by ZIP. **O.** In vitro tumour sphere formation ability of PARD3 OE/wild-type CD133^+^ cells treated with ZIP, as determined by an in vitro limiting dilution assay. *P < 0.05; **P < 0.01; ***P < 0.001; n.s., not statistically significant

Previous study suggested that deregulation of PARD3 may potentially contribute to the epithelial-mesenchymal transition (EMT). Contradictorily PARD3 could either promote or suppress EMT in different types of human cancers [20, 23, 24]. As EMT play an important role in mediating cancer cell stemness, we first evaluated if EMT was regulated in the PARD3-driven hepatocarcinogenesis, Nonetheless, through a re-evaluation of the histological sections obtained from orthotopic tumours in both wild type and PARD3 overexpressing models, we observed that overexpression of PARD3 had marginal effects on the expression of epithelial marker E-cadherin and mesenchymal markers including N-cadherin and vimentin (Fig. S5A). This observation in turn suggested that PARD3 doesn’t promote liver cancer stemness by triggering EMT and alternative signalling pathways are involved in the regulation of PARD3 on cancer stemness. To further characterize the CD133^+^ TICs isolated from PARD3-overexpressing Hepa1-6 cells, we sorted CD133^+^ and CD133^−^ cells from PARD3-overexpressing Hepa1-6 cells and compared their gene expression profiles by RNA-seq (Fig. 4A). Compared to CD133^−^ cells, CD133^+^ cells exhibited differential expression of genes related to multiple cellular events and signal transduction (Fig. 4B). KEGG enrichment analysis revealed that the differentially expressed genes in CD133^+^ cells were related to SHH signalling and PPAR signalling (Fig. 4C). Following a reanalysis of the scRNA-seq data, we found that cancer cells of clusters 0 and 1 with higher stemness potential had more active Hedgehog signalling than those of cluster 2, which was characterized as differentiated cancer cell lineage with lower expression of CD133 and PARD3 (Fig. 4D&E). This observation was consistent with the bulk RNA-seq results. Moreover, in line with the observed augmented SHH signalling activity, gene set enrichment analysis (GSEA) showed that SHH signalling was activated in CD133^+^ cells compared to their CD133^−^ counterparts (Fig. 4F). In stem cells, SHH signalling can contribute to the maintenance of pluripotency and prevent premature differentiation. Similarly, in cancer stem-like cells, the activation of SHH signalling helps sustain their stemness properties, allowing them to self-renew and resist differentiation, thereby promoting tumour growth and heterogeneity [25]. The preferential activation of SHH signalling in CSCs was also documented by previous study [26, 27]. To confirm our results, we sorted CD133^+^ cells and their CD133^−^ counterparts by FACS. The mRNA expression of Gli1, PTCH1 and SHH was significantly higher in CD133^+^ cells (Fig. 4G) and the protein expression of SHH and Gli1 was induced in CD133^+^ cells (Fig. 4H). Immunofluorescence staining on liver tumour tissue sections corroborated that PARD3 overexpression resulted in more profound activation of SHH signalling (Fig. S5B). These observations confirmed that PARD3 overexpression upregulated SHH signalling, which led to the formation of a CD133^+^ cell population capable of tumorigenesis in vivo.

### SHH signalling was responsible for the increased tumorigenicity of PARD3-overexpressing CD133^+^ TICs

Activation of SHH signalling was previously reported in TICs [28]. To examine whether SHH signalling activation is responsible for the PARD3-induced generation of CD133^+^ TICs, We treated Hepa1-6 cells with a pharmacological inhibitor, GANT58, in the presence or absence of PARD3 overexpression. The presence of GANT58 significantly suppressed the generation of the CD133^+^ population within PARD3-overexpressing Hepa1-6 cells (Fig. 4I). The results of an in vitro limiting dilution assay revealed that the presence of GANT58 significantly suppressed the in vitro sphere formation of CD133^+^ TICs isolated from PARD3-overexpressing Hepa1-6 cells (Fig. 4J). Moreover, the expression of stemness-associated genes, including SOX2, Oct4 and Nanog, in PARD3-overexpressing Hepa1-6 cells was inhibited by the presence of GANT58 (Fig. S5C). Further ChIP assays revealed that Gli1 could directly bind to the transcriptional promoter region of the SOX2 gene in PARD3-overexpressing CD133^+^ Hepa1-6 cells, which in turn resulted in SOX2 overexpression and subsequent expression of the key pluripotency factors Oct4 and Nanog [29] (Fig. S5D). These observations confirm the important role of SHH activation in mediating PARD3-induced stemness maintenance in Hepa1-6 cells.

The activity of Gli1 as a transcription factor is regulated by several intracellular signalling mediators, including aPKC, the previously documented downstream signalling target of PARD3 [30]. Consistent with this observation, an interaction between aPKC and PARD3 was detected in PARD3-overexpressing CD133^+^ cells, leading to an increased level of phospho-aPKC (Fig. 4K&L). To identify whether aPKC activity is necessary for Gli1-induced transcription of the SOX2 gene, we treated PARD3-overexpressing CD133^+^ cells and their CD133- counterparts with the aPKC inhibitor ZIP. The presence of ZIP significantly reduced the activation of SHH signalling, as indicated by the reduced expression of its transcriptional products, as well as the cellular expression of Gli1 (Fig. S5E&F), and it decreased the binding capacity of Gli1 with the promoter region of SOX2 (Fig. 4M). In turn, inhibition of aPKC signalling resulted in a reduced CD133^+^ population within PARD3-overexpressing Hepa1-6 cells as well as an attenuated ability of in vitro sphere formation in the limiting dilution assay (Fig. 4N&O). These observations showed that aPKC/SHH signalling is necessary for the acquisition of stemness properties via PARD3 overexpression.

### Clinical significance of PARD3 in TICs

**Fig. 5:**
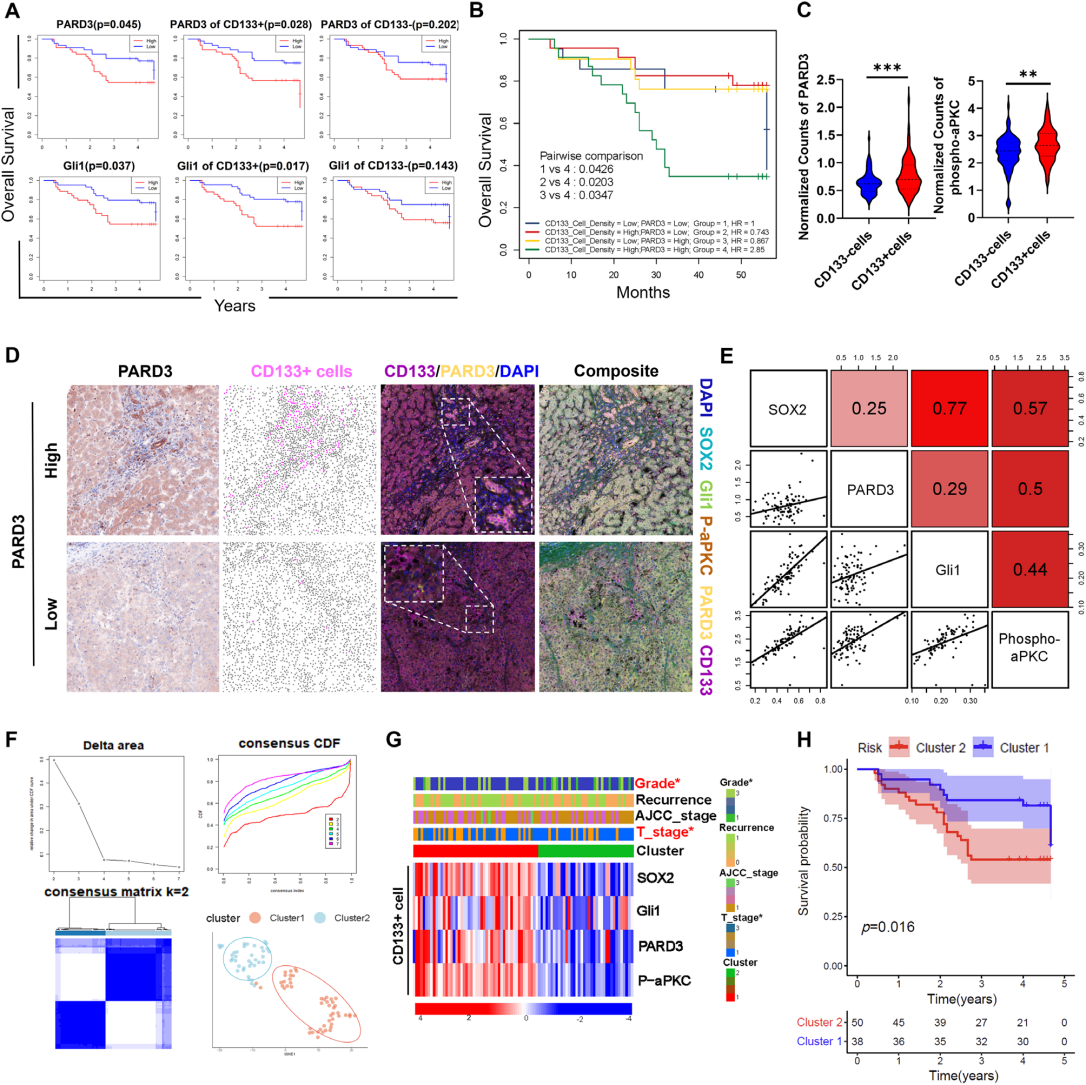
Clinical significance of PARD3, as revealed by multiplex IHC of a tissue microarray. **(A)** KM survival curve showing the prognostic value of overall PARD3 and Gli1 expression and their expression in CD133 + cells and CD133- cells. **(B)** Patients harbouring a high density of CD133 + cells with high PARD3 expression had the worst prognosis relative to patients in the other subgroups. **(C)** The levels of PARD3 and phospho-aPKC were significantly higher in CD133 + cells than in CD133- cells. **(D)** Representative image showing that higher expression of PARD3 was associated with higher presence of CD133 + stem like cancer cells. **(E)** Correlation heatmap showing that the expression levels of PARD3/aPKC/Gli1/Sox2 were positively intercorrelated in CD133^+^ cells. **(F)** Consensus clustering tools were used to classify the 88 patients into two subtypes as determined by setting k to 2. **(G)** Patients in cluster 1 exhibited higher activation of PARD3/aPKC/Gli1/Sox2 in CD133^+^ cells, accompanied by less favourable clinicopathological features and **(H)** worse overall survival. *P < 0.05; **P < 0.01; ***P < 0.001; n.s., not statistically significant

To explore the clinical significance of our findings, we performed multiplex IHC on a tissue microarray containing 88 HCC tissues and paired normal liver tissues. The procedure for whole-slide staining and the overall workflow are shown in Fig. S6. To separately quantify target gene expression in TICs in the tissue sections, we adopted a training-based algorithm (inForm software for Akoya Vestra Polaris) to automatically label each cell with a phenotype tag. In this system, CD133^+^ cells were assigned to TICs, visualized as pink dots, and their CD133^−^ counterparts were visualized as brown dots (Fig. S6). Representative images of tumour and adjacent liver tissues were shown in Fig. S7A. Then, Kaplan–Meier (KM) analysis was conducted to investigate the potential prognostic roles of the key molecules involved in this study. Significantly worse prognosis was observed in patients with high expression of PARD3 and Gli1, and the expression of PARD3 and Gli1 in CD133^+^ cells more accurately predicted prognosis than did that in CD133^−^ cells (Fig. 5A). The high density of CD133^+^ cells, although exhibiting no significant value in KM analysis, distinguished patients with the worst prognosis when combined with the high level of PARD3, as revealed in KM analysis of patient subgroups (Fig. 5B). There was no significant difference in the expression of PARD3 observed between patients with and without hepatitis virus infection. However, patients with advanced stage HCC exhibited a significantly higher expression of PARD3 (Fig. S7B). The levels of both PARD3 and phospho-aPKC were significantly higher in CD133^+^ cells than in CD133^−^ cells (Fig. 5C), suggesting that PARD3 exerts a more important signal transduction function in CD133^+^ cells. Additionally, representative images showed that PARD3 and Gli1 exhibited robust colocalization with CD133 (Fig. S7C). Patients with higher level of PARD3 were associated an increased presence of CD133^+^ stem like cancer cell which was denoted as pink dots (Fig. 5D and S7D), further underlining the important role of PARD3 in TICs expansion. Pearson correlation analysis showed that the expression levels of most signalling molecules evaluated in this study were positively intercorrelated in CD133^+^ cells (Fig. 5E). The most significant correlation was observed between Sox2 and Gli1, consistent with the results of our ChIP assay. In addition, the phosphorylation of aPKC was significantly correlated with the expression of Gli1, PARD3 and Sox2 (*R* > 0.4), emphasizing the important role of aPKC in mediating PARD3, SHH signalling and cancer stemness. Additionally, we stratified the HCC patients into two clusters by using the *consensus clustering* R package, in which the cumulative distribution function (CDF) delta area curve exhibited relatively stable clustering when the cluster number was set to be 2 (Fig. 5F). The patients in cluster 2 showed significantly higher activation of PARD3/aPKC/Gli1/Sox2 axis in CD133^+^ cells and had higher histological grades and T stages than their counterparts in cluster 1 (Fig. 5G). Consistent with this finding, patients in cluster 2 with higher PARD3 mediated cancer stemness activity exhibited significantly worse overall survival (Fig. 5H). These results demonstrate that the PARD3/aPKC/Gli1/Sox2 axis in CD133^+^ cells has potential clinical significance in predicting the prognosis of HCC patients.

### Berberine suppressed HCC tumorigenesis by inhibiting PARD3 expression

We then tested the antitumorigenic property of the natural compound berberine, which is isolated from the medicinal plant *Coptidis rhizoma* and has long been used as an over-the-counter medicine for gastrointestinal diseases [31]. To systematically evaluate the prophylactic activity of berberine, we started berberine intervention (10 mg/kg/3 days, i.p.) at different stages of the liver disease spectrum in MCD diet-fed mice (Fig. S8A). Interestingly, initiation of berberine treatment at different stages of liver diseases exhibited no significant difference in preventing or treating hepatic inflammation and fibrosis (Fig. S8B); however, treatment initiation at any stage potently prevented tumour formation, decreased the tumour multiplicity and tumour volume in the end-stage livers of MCD diet-fed mice (Fig. S8C-S8E). The serum AFP level in MCD diet-fed mice was significantly reduced by berberine treatment (Fig. S8F). Moreover, no significant changes in the histological features of major organs were observed in the mice upon berberine treatment (Fig. S8G).

**Fig. 6:**
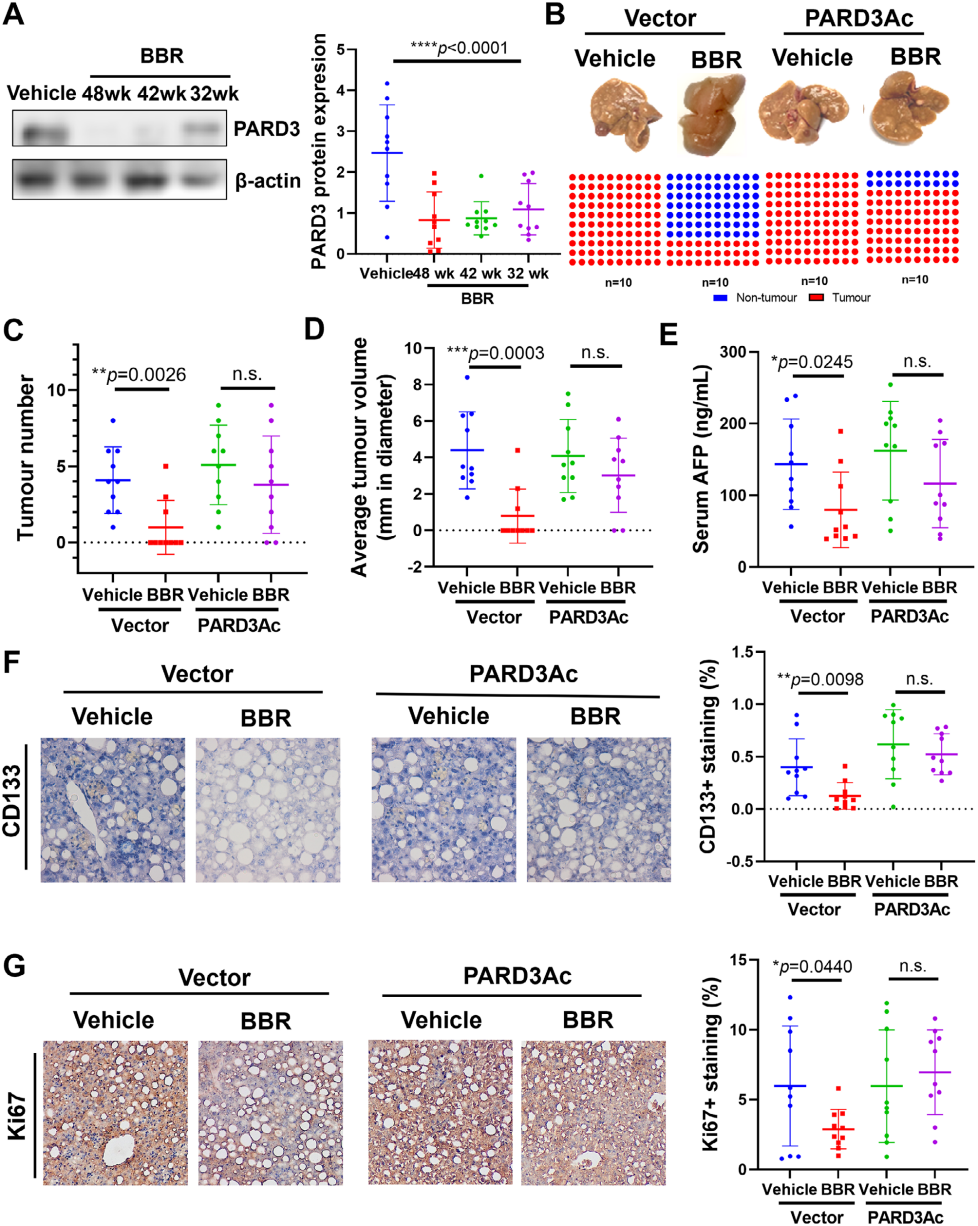
Berberine suppressed HCC tumorigenesis by inhibiting PARD3 expression. **(A)** Berberine suppressed PARD3 expression in the liver. Restoration of PARD3 expression abolished the effects of berberine and increased the **(B)** tumour incidence, **(C)** tumour multiplicity, **(D)** tumour volume and **(E)** serum AFP level. **F&G.** Recovery of PARD3 effectively abolished the inhibitory effects of berberine on Ki67 and CD133, as measured by immunohistochemical analysis. *P < 0.05; **P < 0.01; ***P < 0.001; n.s., not statistically significant

Suppression of PARD3 expression in the liver was observed (Fig. 6A). To identify the role of PARD3 inhibition in mediating the suppression of tumorigenesis by berberine treatment, we restored PARD3 expression in the livers of mice treated with berberine. Restoration of PARD3 expression significantly increased the risk of tumorigenesis in berberine-treated mice (Fig. 6B) and increased the tumour multiplicity, tumour volume and serum AFP level (Fig. 6C-E). Immunohistochemical analysis revealed that berberine treatment potently reduced the cellular expression of Ki67 as well as the CD133^+^ population in the livers of mice, while restoration of PARD3 expression abolished the inhibitory effect of berberine on Ki67 and CD133 expression (Fig. 6F&G). These observations indicate that berberine may be useful as a prophylactic intervention against hepatic tumorigenesis in patients with liver diseases by targeting PARD3.

## Discussion

In this study, we identified PARD3 as the critical initiator of hepatic tumorigenesis in mouse models of NAFLD/nonalcoholic steatohepatitis (NASH). PARD3 upregulation promoted self-renewal activity in hepatic TICs and therefore facilitated tumorigenesis in vivo. Interestingly, in the early stages of hepatic disease progression, there were no significant changes in the expression of PARD3, with PARD3 upregulation occurring only at the tumorigenesis stage. This pattern indicated that the inflammatory and metabolic stress introduced in the early stages did not contribute to the induction of PARD3 expression in the cells. A previous study reported that the expression of PARD3 was more strongly induced in human HCC tissue than in nontumor normal liver tissue, a pattern possibly related to genomic instability, based on gene association analysis [32]. This finding suggests that PARD3 gene mutation may play a role in its upregulated expression in HCC. Deleterious mutations of PARD3 have been identified in several human diseases, including cancers. Homozygous deletion of PARD3 was observed in human oesophageal squamous cell carcinoma [33]. A rare deleterious variant of PARD3 was found to be significantly associated with an increased risk of cranial neural tube defects in patients [34]. To further investigate the underlying mechanisms leading to PARD3 overexpression in HCC tissues, we first investigated the epigenetics alternations of PARD3 in the TCGA-LIHC dataset by virtue of DNMIVD database [35], yet we didn’t observe any significant changes in the DNA methylation on the promoter region between normal and tumour groups, suggesting that the dysregulation of PARD3 may be not attributed to this epigenetic mechanism (Fig. S9A). On the other hand, as liver cancer is known to exhibit a complex genomic landscape, and copy-number alterations (CNAs) are frequently observed, these CNAs can contribute to the dysregulation of genes involved in tumor development, progression, and metastasis. Therefore, we retrieved the CNAs data of TCGA pancancer datasets in the cBioPortal database (https://www.cbioportal.org/). In the case of PARD3, copy-number gains, which refer to an increased copy number of the genomic region encompassing the PARD3 gene compared to the normal state, were one of the most frequently observed CNAs in liver cancer (Fig. S9B). The CNAs characterized as “gains” or “amplification” result in an elevated number of copies of the PARD3 gene in the cancer cells’ genome, leading to higher levels of PARD3 mRNA and protein in liver cancer cells (Fig. S9C). Therefore, we speculate that CNAs likely play a role in the upregulation of PARD3 and may contribute to the neoplastic behaviour observed in liver cancer. Additionally, it was previously found that PARD3 amplification was acquired in radiation-exposed human retinal pigment epithelial cells, leading to increased transcription of PARD3 and contributing to neoplastic transformation of normal epithelial cells [36]. These findings supported our hypothesis that PARD3 overexpression at the tumour initiation stage of hepatic diseases, which is probably due to a genetic instability-induced amplification mutation, contributes to tumorigenesis and progression in HCC.

The PARD3 protein was first discovered in the anterior pole of asymmetrically dividing stem cells of *C. elegans* and is evolutionarily conserved across species. In humans, PARD3 is characterized as a regulatory molecule of cell polarity, migration, and tight junction assembly in dynamic protein complexes [37]. In nonepithelial tissue, PARD3 expression is involved in establishing asymmetric division of stem cells, resulting in neuron polarity and T-cell polarization. It also plays a role in stemness maintenance in neuron progenitor cells, whereas loss of PARD3 promotes neuronal differentiation [38–40]. Although PARD3 expression is differentially regulated in human cancers, and PARD3 may function as either an oncogene or tumour suppressor depending on cancer subtype or stage [22], a recent study showed that in human glioblastoma, PARD3 was enriched in NESTIN-positive stem cells. Similar to our findings, silencing PARD3 was found to lead to a defect in glioma sphere formation in vitro, suggesting that the self-renewal capacity of TICs was lost upon PARD3 silencing [41]. By single-cell analysis, we showed that in an early established HCC tumour, PARD3 was enriched in cancer cells expressing CD133, the cell surface marker of liver cancer stem cells. Subsequent pseudotime analysis of the cancer cell population showed that PARD3 was preferentially enriched in cancer cells at early developmental stages but not in those at differentiated stages, further indicating the essential role of PARD3 as a driver of self-renewal maintenance in TICs. In differentiated epithelial-like cancer cells, loss of PARD3 has been extensively reported to regulate HIPPO signalling for the expansion of the cell population by directly interacting with and dephosphorylating YAP1 via its PDZ domain, which is essential for YAP1 activation [42, 43]. While the expansion of differentiated cancer cells upon PARD3 loss can lead to tumour enlargement, contradictory findings indicating that PARD3 overexpression promoted tumour progression in vivo have been obtained [44]. Our findings showed that PARD3 expression in hepatic TICs promoted their self-renewal and in vivo tumorigenicity, suggesting a different role and mechanism of PARD3 in undifferentiated stem-like cells. We indeed did not observe activation of HIPPO/YAP signalling upon PARD3 overexpression in TICs, suggesting that HIPPO signalling may not serve as a critical mediator of PARD3-induced self-renewal in TICs. A previous study revealed that PARD3 may play diverse roles in regulating HIPPO/YAP signalling activity in different types of cells [42]. By RNA-seq analysis, we found that the SHH pathway may be the downstream signalling pathway of PARD3 in hepatic TICs by driving transcriptional activation of stemness-associated genes. Moreover, inhibition of SHH activation abolished PARD3-induced maintenance of self-renewal in hepatic TICs. Preferential activation of SHH in TICs could be possibly due to the distinct gene expression profile of cancer stem-like cells from that of differentiated cancer cells within a heterogenous tumour tissue. PARD3 is reported to be intrinsically associated with stem cell maintenance and self-renew [22]. The preferential activation of SHH signalling by PARD3 in stem like cancer cells could contribute to the maintenance of their stem-like state, promoting self-renewal and inhibiting differentiation. Interestingly, our data and previous study suggested that PARD3 is differentially expressed in stem-like cancer cells and differentiated cancer cells. Mahsa et al. reported PARD3 protein enrichment in SOX2-, CD133-, and NESTIN-positive stem like glioblastoma cells [41]. Our scRNA sequencing analysis demonstrated a positive correlation between the expression of PARD3 and CD133 in the cancer stem cell lineage with highly activated SHH signalling (Fig. 3D). Furthermore, our multiplex immunohistochemistry (IHC) analysis conducted on a tissue array provided additional support for this notion, as evidenced by the elevated signal intensity of PARD3 and phosphorylated aPKC in CD133 + cells (Fig. 5C), which in turn underlines the important role of PARD3 in regulating SHH signalling in CD133 + TICs. These results further underline the critical role of PARD3 in the modulation of SHH signalling within the CD133 + TIC population.

Berberine is a natural alkaloid isolated from the Chinese medicinal herb *Coptidis rhizoma* that exhibits potent anticancer activities. Previous studies by our laboratory revealed that berberine exhibited dose-dependent inhibitory activity against HCC. A cytotoxic dose of berberine suppressed the proliferation [45] and induced the death of HCC cells [46], whereas low-dose berberine treatment suppressed tumour angiogenesis and invasion of HCC cells [47]. Berberine also suppressed tumour growth and lung metastasis in vivo [47]. Mechanistically, berberine regulates oncogenic and tumour suppressor genes in HCC through transcriptional [47], posttranscriptional [45] and epigenetic [48] mechanisms. Berberine was also reported to suppress TIC activities in breast cancer, oral carcinoma and ovarian cancer [49–51]. Notably, the activation of Gli1 by chemotherapy in ovarian TICs can be significantly repressed by the presence of berberine [49]. We found that berberine potently suppressed PARD3, whose expression was essential for activation of the Gli1 and SHH pathways, consistent with the role of berberine in regulating TICs in HCC tumorigenesis. From a clinical perspective, berberine is an over-the-counter medicine in China that has been used for several decades for the treatment of gastrointestinal inflammation. Interestingly, a recent multicentre, double-blind, placebo-controlled clinical trial revealed that berberine can effectively prevent the recurrence of colorectal cancer after complete polypectomy, suggesting that berberine may be a well-tolerated and effective treatment for cancer prevention [52]. Our study proved that berberine suppressed experimental hepatic tumorigenesis by targeting PARD3. However, determining the efficacy and safety of berberine for HCC prevention requires further clinical investigation.

PARD3’s involvement in cellular processes related to tumorigenesis makes it an attractive target for preventive strategies. Inhibiting PARD3 activity could potentially impede the transformation of normal liver cells into cancer cells, thereby preventing tumorigenesis. This preventive approach holds promise for high-risk individuals, such as those with preexisting liver conditions or genetic predispositions. However, it is important to consider the potential off-target effects or unintended consequences of targeting PARD3, which also plays an important role in normal cell development [39, 53, 54]. Ensuring efficient and selective delivery to tumour cells while minimizing exposure to normal liver cells is crucial. Yet delivering the targeted inhibition agents specifically to liver cancer cells can be challenging in the field of targeted therapeutics. Besides, cancer cells can develop resistance mechanisms against targeted therapies over time, especially given liver cancer is a heterogeneous disease, both at the molecular and cellular levels. In the future, it would be of great interest to explore the potential of targeted PARD3 inhibition as a combinatorial therapeutic approach, to assess its synergistic effects when combined with chemotherapy and immunotherapy. Besides, our tissue array multiplex IHC analysis showed that PARD3 was associated with cancer stemness and more aggressive tumor progression. By incorporating PARD3 evaluation into clinical practice, physicians can better stratify patients, predict disease progression and adjust treatment plan accordingly.

**Fig. 7:**
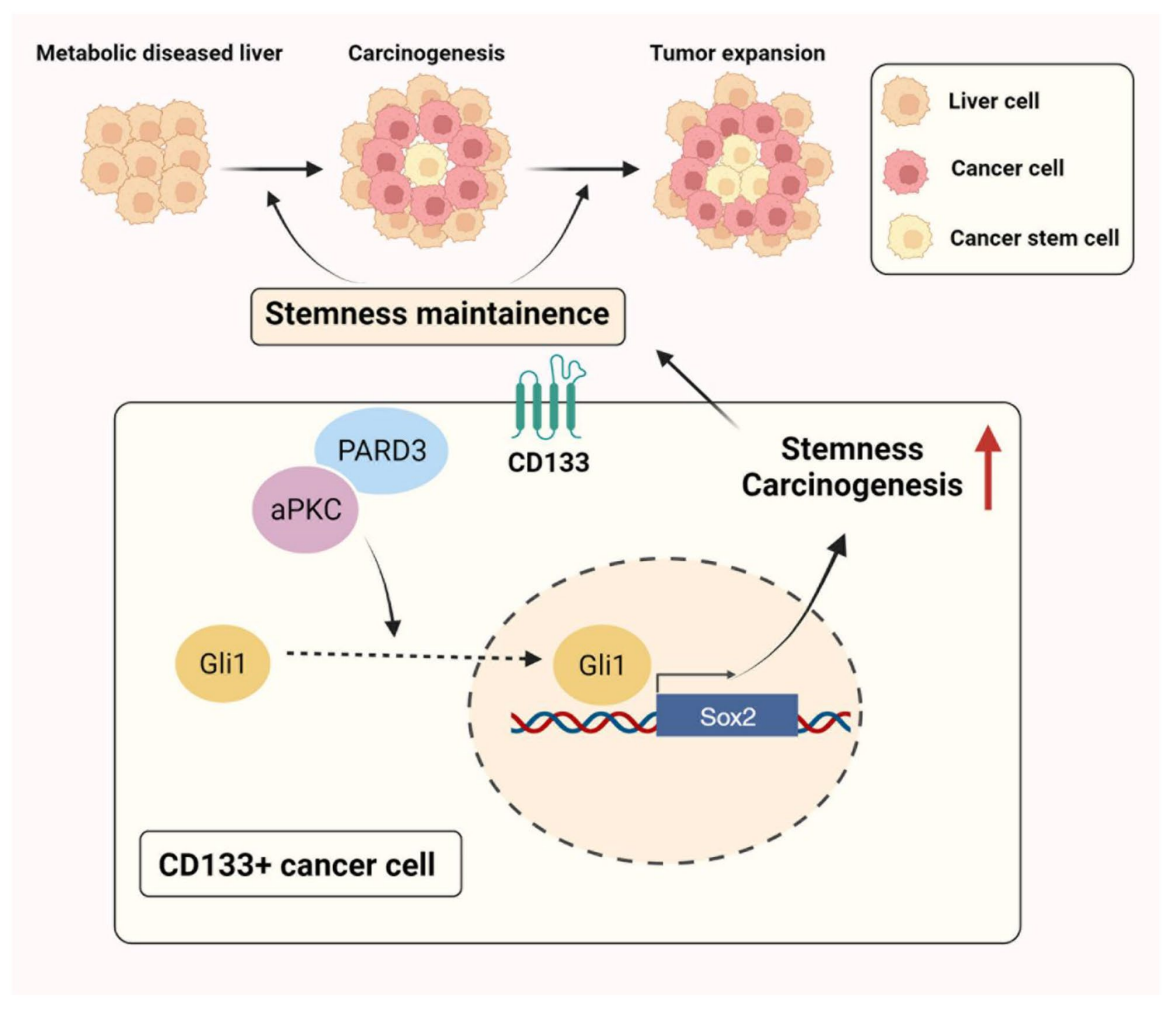
Mechanisms involved in the regulation of PARD3 on TICs in hepatocarcinogenesis

In conclusion, in this study, we identified PARD3 as an important initiator that facilitates hepatic tumorigenesis. PARD3 was overexpressed in HCC and predicted poor prognosis in patients. Upregulation of PARD3 was observed specifically at the tumorigenesis stage in spontaneous liver tumour models, and knockdown of PARD3 inhibited hepatic tumorigenesis, while forced PARD3 expression accelerated cancer progression. Single-cell analysis revealed that PARD3 overexpression resulted in the enrichment of a stem-like CD133^+^ cell population in HCC and helped to maintain the self-renewal ability of these cells by activating SHH signalling and the transcription of downstream stemness-associated genes (Fig. 7). Clinicopathological analysis showed that the expression of PARD3 was positively associated with the expression of SHH signalling molecules, which together could be used to stratify HCC patients into two clusters with different clinical characteristics and overall survival outcomes. As a candidate drug targeting PARD3, berberine potently prevented hepatic tumorigenesis. Our study elucidated the role of PARD3 as a targetable molecule for the prevention of hepatocarcinogenesis.

### Electronic supplementary material

Below is the link to the electronic supplementary material.

Supplementary Material 1

## Data Availability

The datasets used and/or analysed during the current study are available from the corresponding author on reasonable request.

## Declarations

Protocols of all the animal studies were reviewed and approved by the Committee of Use of Laboratory Animal in Teaching and Research (CULATR) in the University of Hong Kong.
