# Supplementary material for: PARD3 drives tumorigenesis through activating Sonic Hedgehog signalling in tumour-initiating cells in liver cancer
Source: J Exp Clin Cancer Res. 2024 Feb 6;43:42. doi: 10.1186/s13046-024-02967-3 (PMC10845773; doi:10.1186/s13046-024-02967-3)
Supplement: Supplementary file 1 — Supplementary Material 1 [file 13046_2024_2967_MOESM1_ESM.docx]

**Supplemental Materials**

**Supplemental Figures**

**
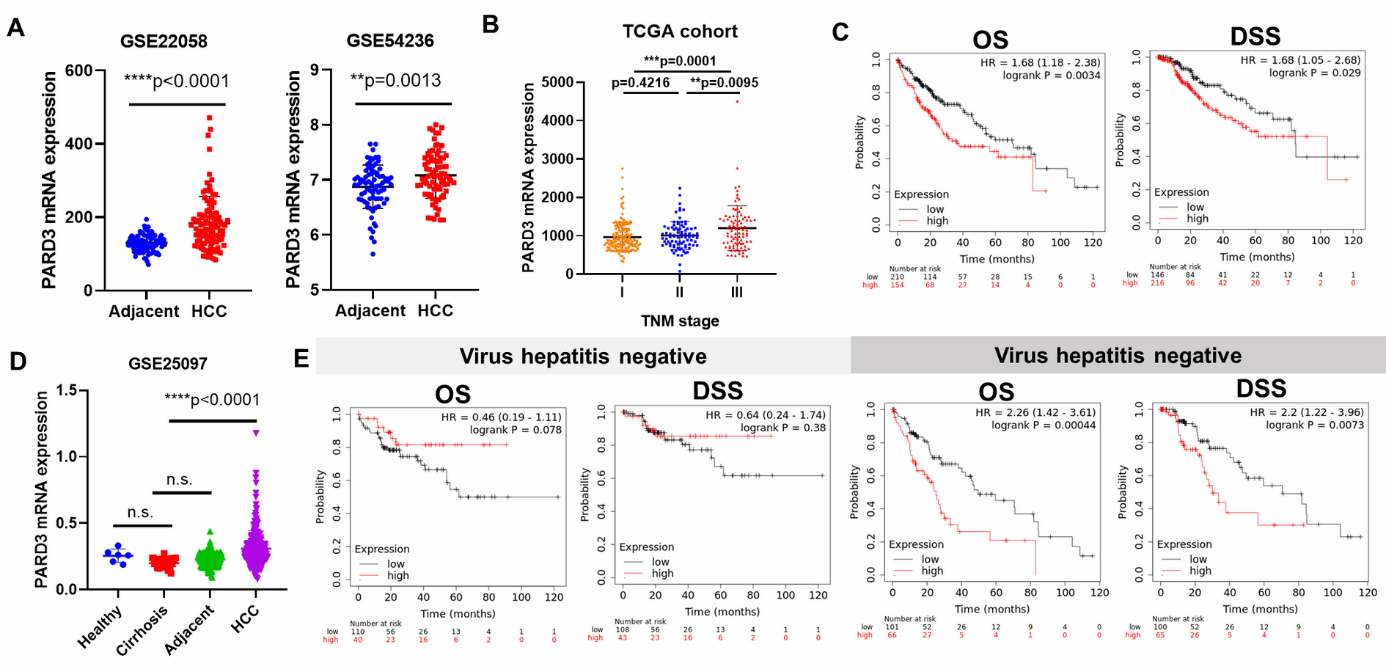
**

**Figure S1 Expression profile of PARD3 in HCC transcriptome datasets. A.** The mRNA expression level of PARD3 in HCC and paired adjacent normal tissues. **B.** The expression of PARD3 in different TNM stages of HCC patients in the TCGA-LIHC dataset. **C.** KM curves showing that high expression of PARD3 was associated with poor overall survival (OS) and disease-free survival (DFS). **D.** Expression of PARD3 in healthy liver, cirrhotic liver, adjacent liver and HCC tissues. **E.** KM curves showing the prognostic significance of PARD3 in hepatitis-negative and hepatitis-positive patients. **P* < 0.05; ***P* <0.01; ****P* < 0.001; n.s., not statistically significant.


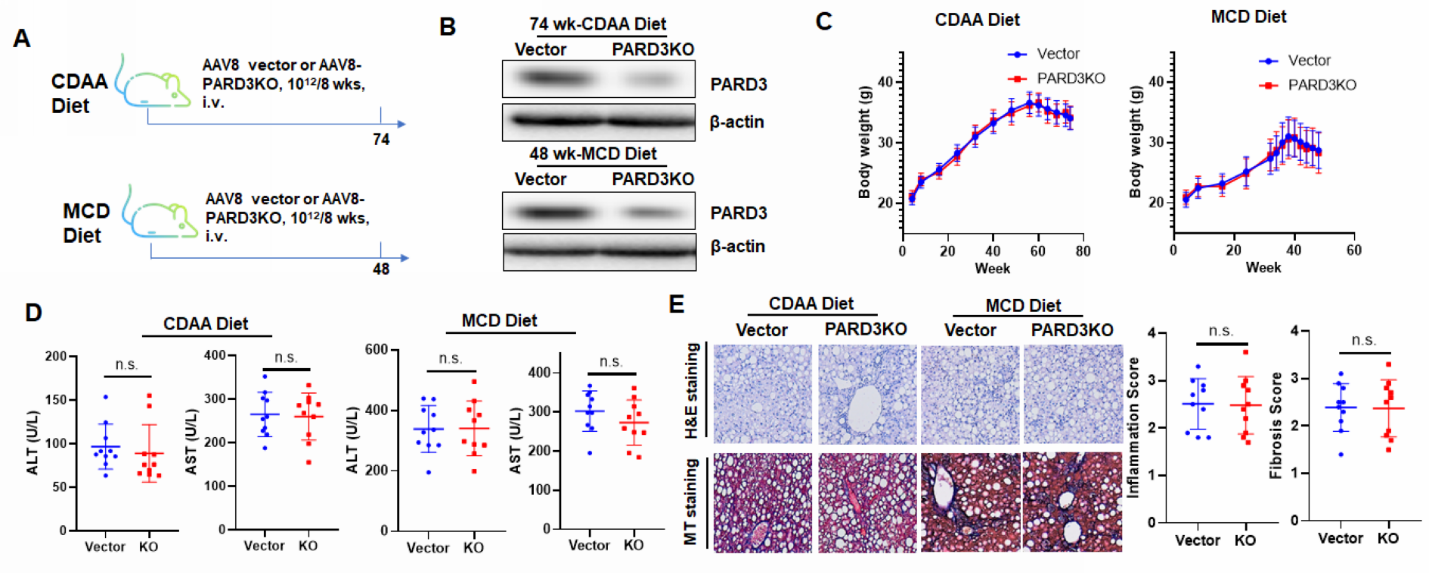


**Figure S2 PARD3 knockout did not induce body weight changes or inflammation. A.** Diagram showing the study design of PARD3 knockout in diet-induced hepatocarcinogenesis. **B.** Successful knockout of PARD3 in the mouse liver. No significant differences in **C.** body weight or **D.** ALT and AST levels were observed between mice with PARD3 knockout and their wild-type littermates. **E.** No significant differences in hepatic inflammation and fibrosis were observed between mice with PARD3 knockout and their wild-type littermates. **P* < 0.05; ***P* <0.01; ****P* < 0.001; n.s., not statistically significant.


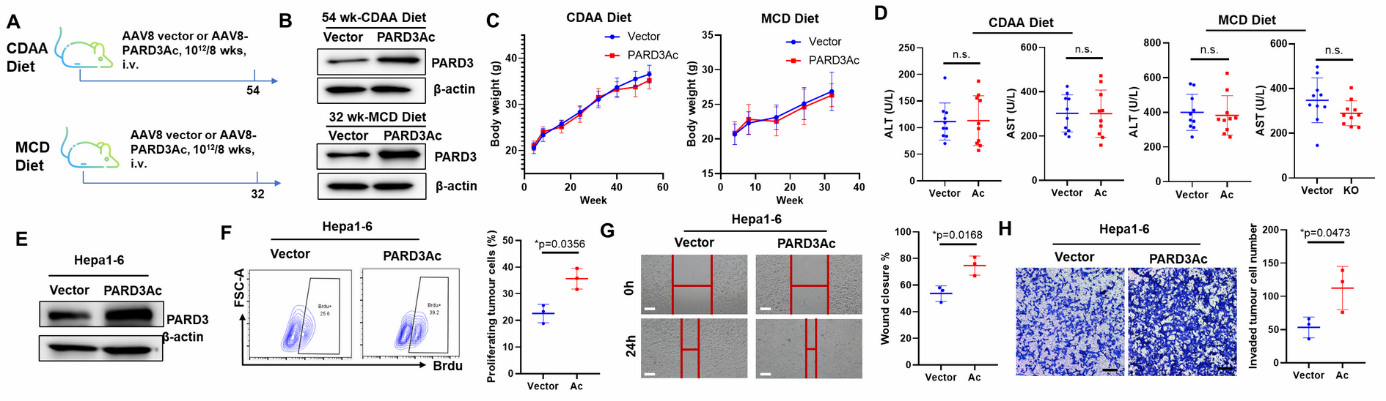


**Figure S3 PARD3 activation promoted liver cancer progression. A.** Diagram showing the study design of PARD3 overexpression in diet-induced hepatocarcinogenesis. **B.** Successful overexpression of PARD3 in the mouse liver, as measured by immunoblotting. No significant differences in **C.** body weight or **D.** ALT and AST levels were observed between mice with PARD3 overexpression and their wild-type littermates. **E.** Stable clones of PARD3-overexpressing Hepa1-6 cells were established, and PARD3 overexpression was measured by immunoblotting. PARD3 overexpression resulted in significantly increased **F.** proliferation, **G.** migration and **H.** invasion of Hepa1-6 cells. **P* < 0.05; ***P* <0.01; ****P* < 0.001; n.s., not statistically significant.


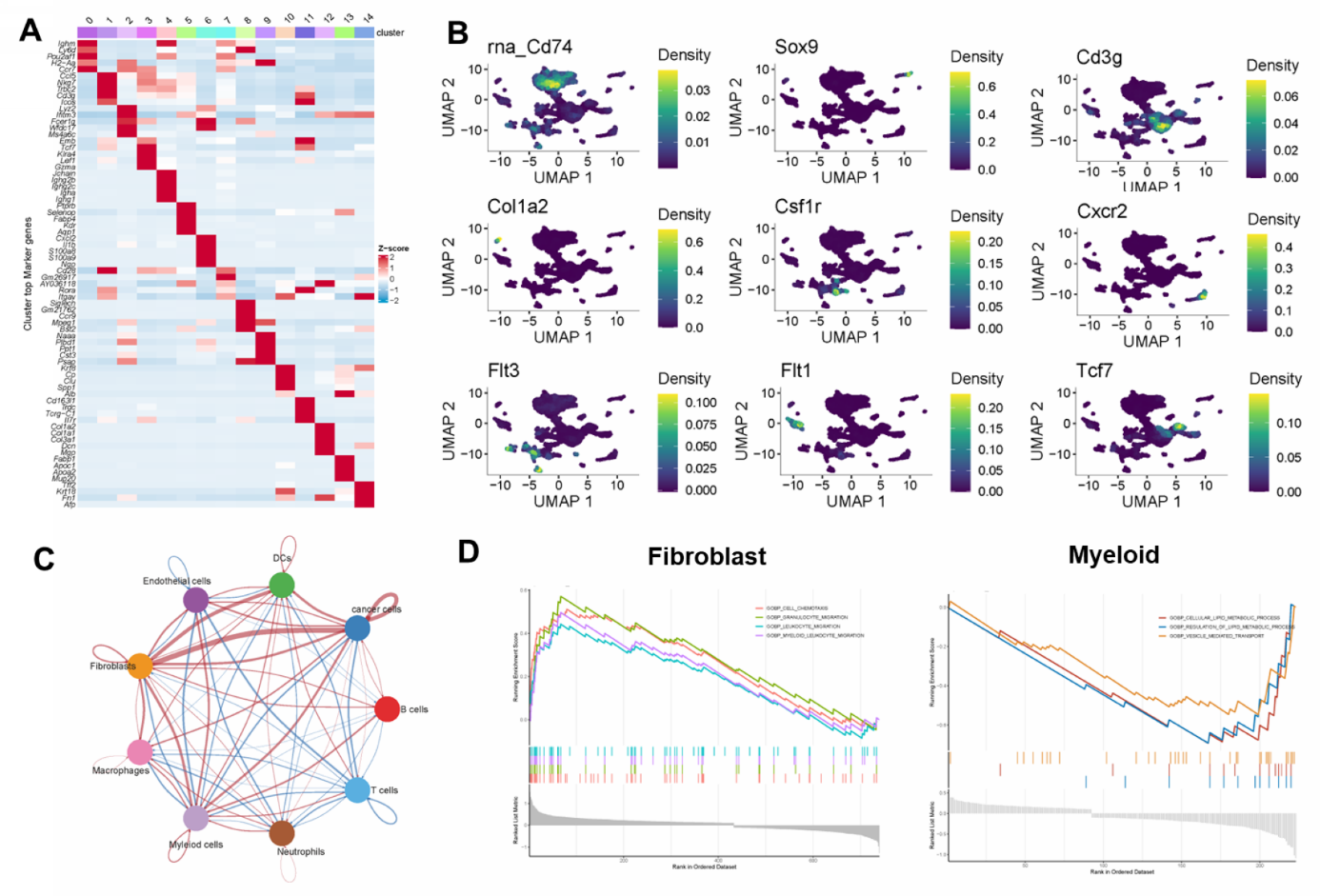


**Figure S4 Single-cell data analysis. A.** Top 5 marker genes of the 15 cell clusters identified. **B.** Cell markers used to annotate different lineages. **C.** Changes in cell‒cell interactions upon PARD3 overexpression, as revealed by CellChat. **D.** GSEA of fibroblasts and myeloid cells.


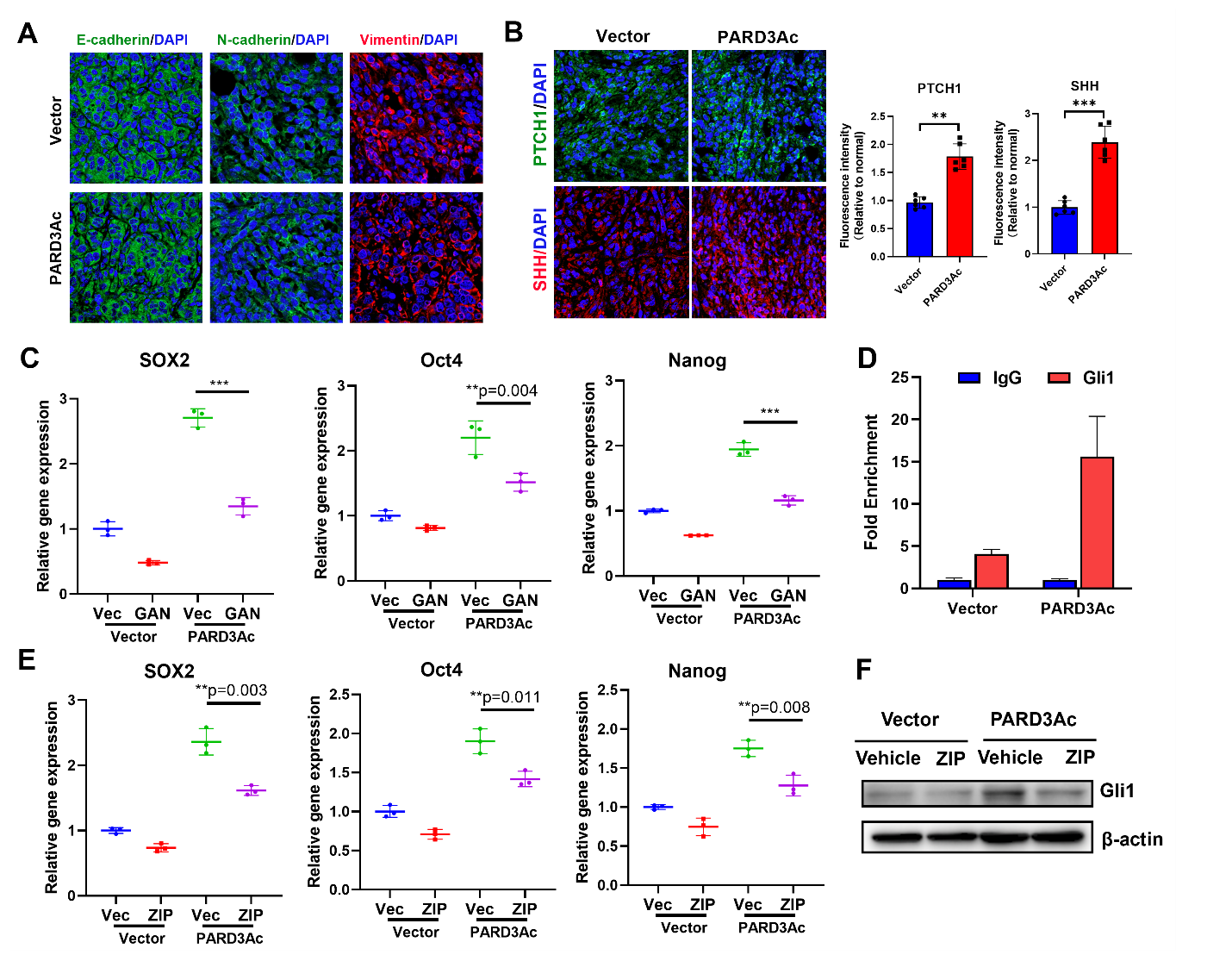


**Figure S5 aPKC was a key mediator of PARD3-induced SHH activation. A.** PARD3 overexpression showed negligible effects on the epithelial-mesenchymal transition (EMT) behaviour of Hepa1-6 orthotopic tumour. **B.** SHH signalling was activated in PARD3 overexpressing tumour section as revealed by SHH and PTCH1 immunofluorescence staining. **C.** Changes in the relative expression of SOX2, Oct4 and Nanog in PARD3 OE/wild-type CD133^+^ cells with or without GANT58 treatment. **D.** Changes in the relative expression of SOX2, Oct4 and Nanog in PARD3 OE/wild-type CD133^+^ cells with or without GANT58 treatment. **E.** Relative expression of SHH signalling-associated genes in PARD3 OE/wild-type CD133^+^ cells upon treatment with ZIP. **F.** Gli1 protein expression in PARD3 OE/wild-type CD133^+^ cells treated with ZIP. **P* < 0.05; ***P* <0.01; ****P* < 0.001; n.s., not statistically significant.


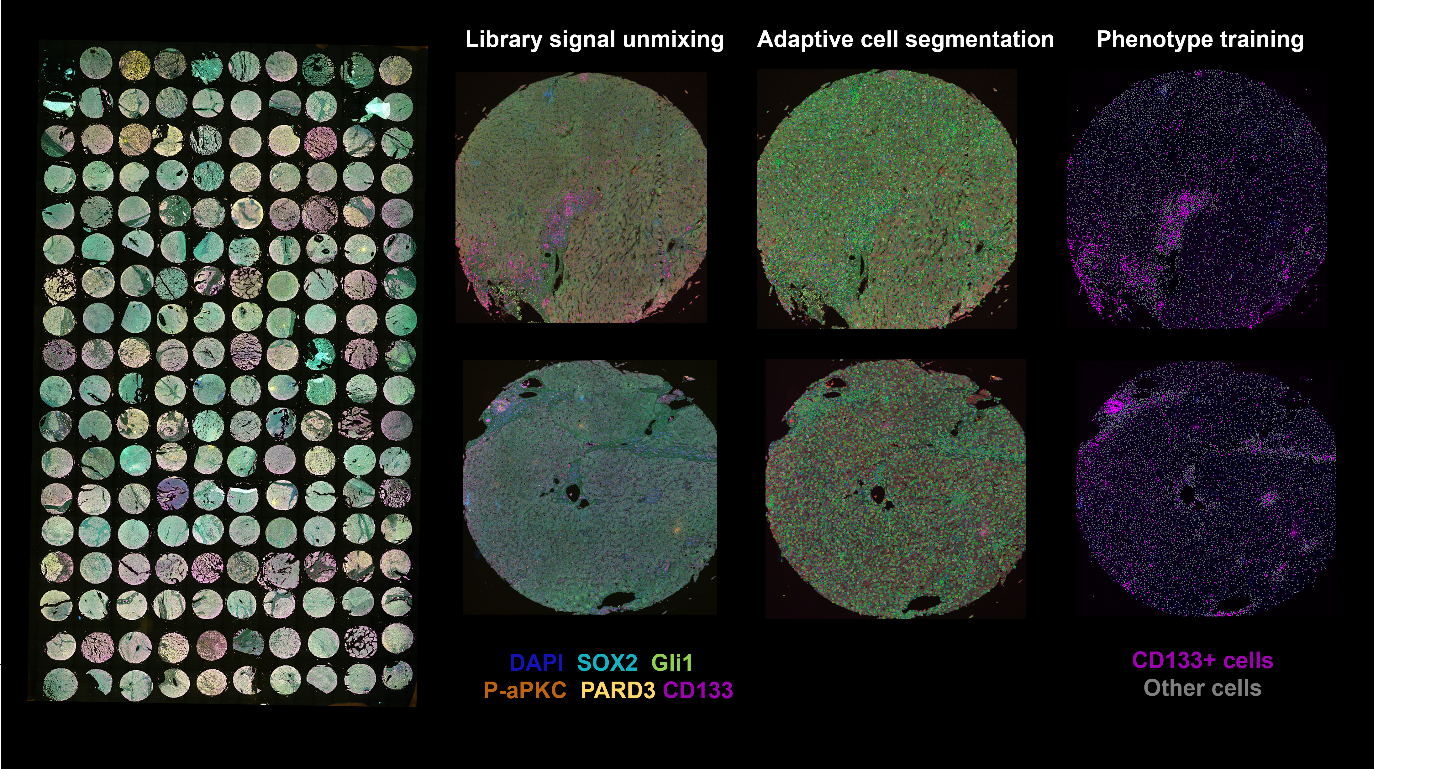


**Figure S6. Whole-slide staining and overall workflow of multiplex IHC of a tissue microarray.**


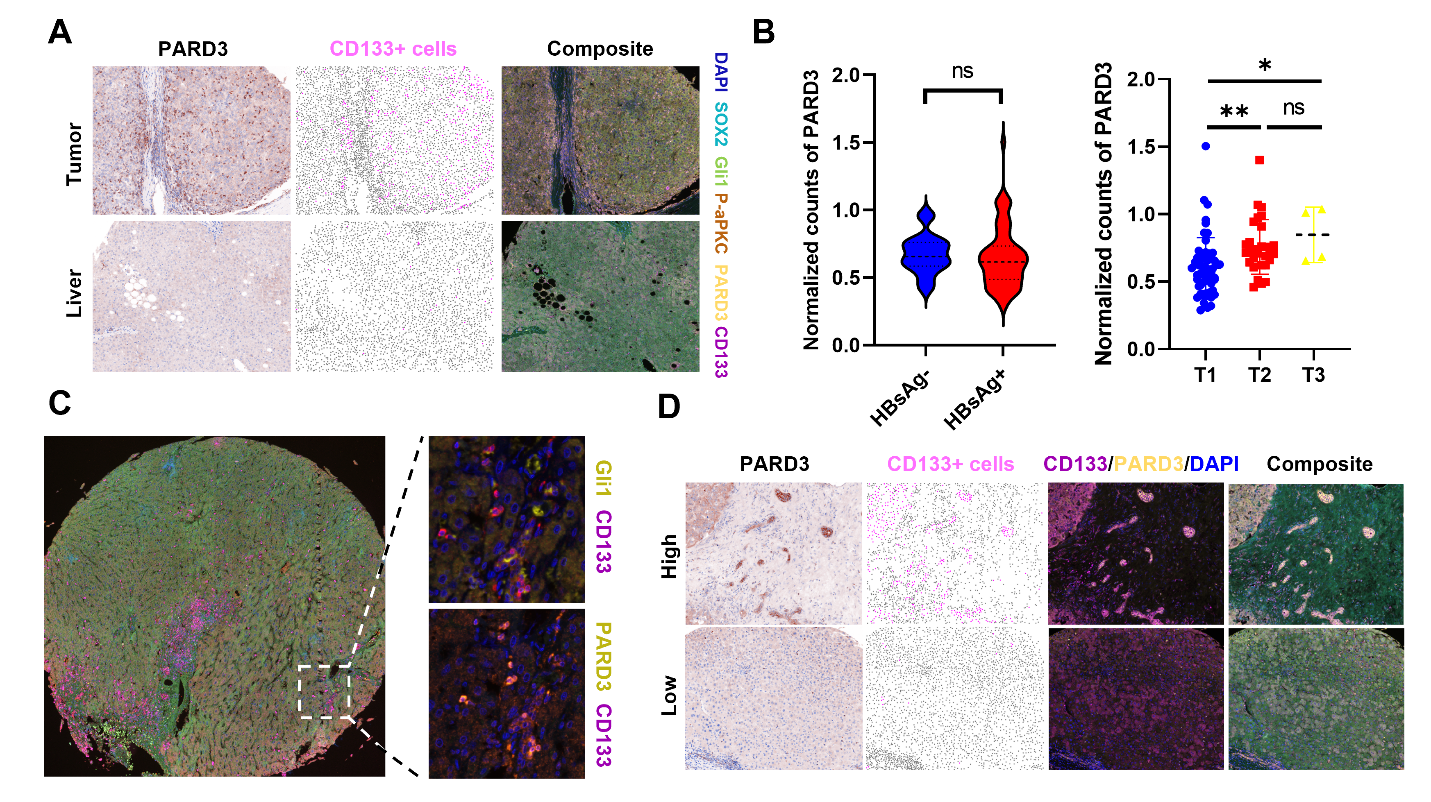


**Figure S7. Representative images of the multiplex IHC performed on tissue microarray. A.** Representative images showing the PARD3 raw IHC staining, CD133^+^ cell phenotype map and composite image of tumour and adjacent liver tissue. **B.** Expression of PARD3 in HBsAg positive and negative patients, and patients of different T stages. **C.** Representative image showing the colocalization of Gli1 with CD133 and PARD3 with CD133. **D.** Higher expression of PARD3 was associated with higher presence of CD133^+^ stem like tumour cells. **P* < 0.05; ***P* <0.01; n.s., not statistically significant.


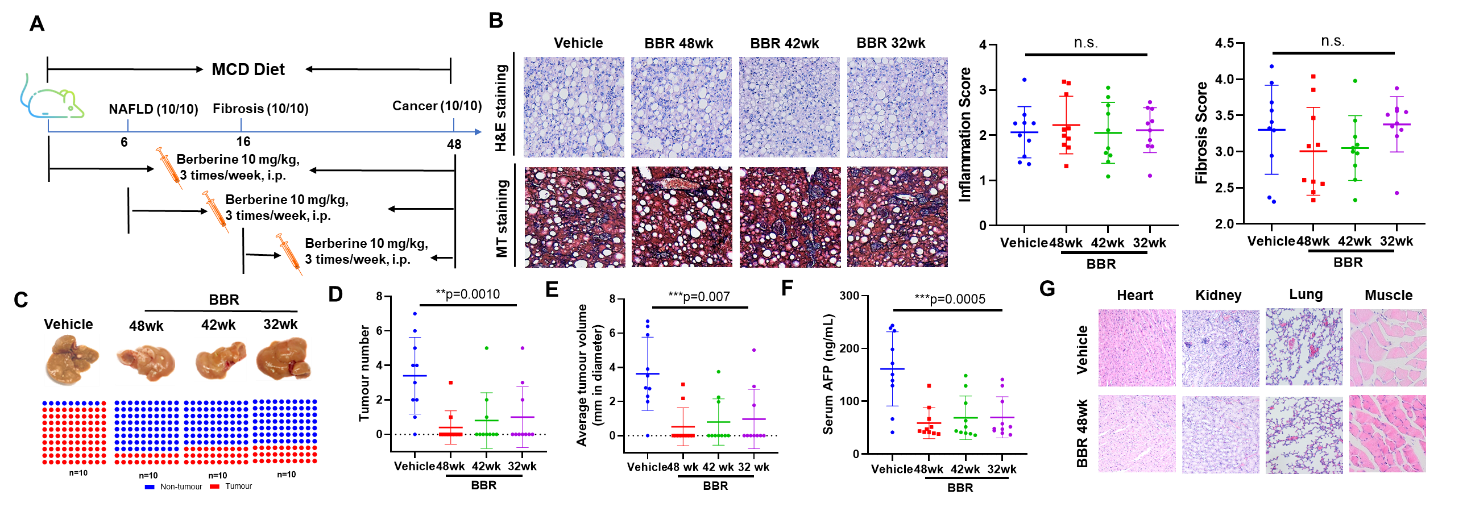


**Figure S8 Berberine suppressed diet-induced hepatocarcinogenesis. A.** Diagram showing the study design. Berberine treatment starting in different stages of liver diseases decreased the **B.** liver tumour incidence, **C.** tumour multiplicity and **D&E.** tumour volume in MCD diet-fed mice. **F.** The serum AFP level was reduced upon berberine treatment. **G.** No significant changes in major organ histology were observed upon berberine treatment. **P* < 0.05; ***P* <0.01; ****P* < 0.001; n.s., not statistically significant.


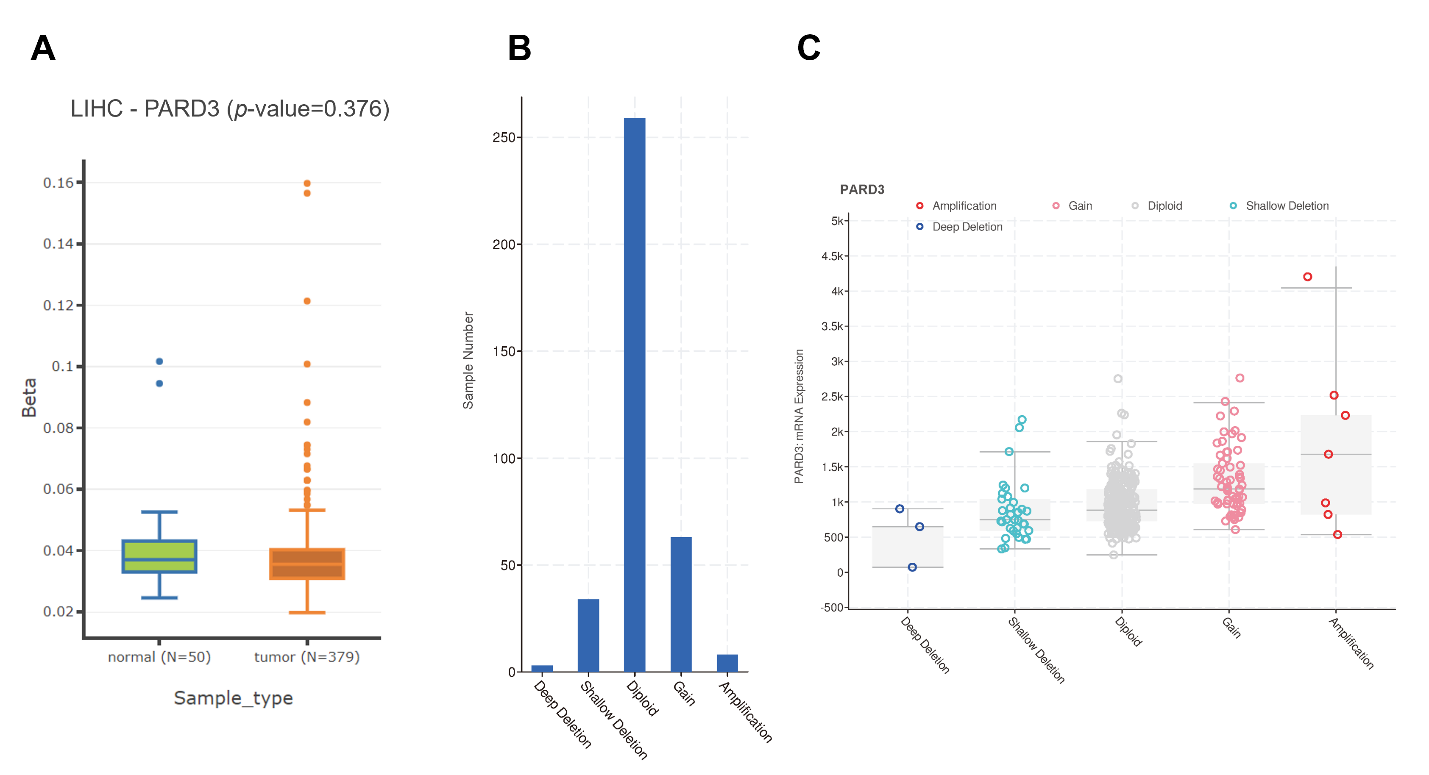


**Figure S9 Potential upstream mechanisms driving PARD3 upregulation during hepatocarcinogenesis. A.** Quantification of DNA methylation of the promoter region of PARD3 in TCGA normal liver and HCC samples. Data was collected in DNMIVD database. **B.** Copy-number gains was the most frequently observed CNAs in liver cancer. **C.** Copy-number gains and amplification result in higher expression of PARD3. Data was collected in cBioportal database.

**Supplemental Tables**

**Table S1 Primer list**

| Gene/Assay | Forward primer (5’-3’) | Reverse primer (5’-3’) |
| --- | --- | --- |
| Gli1 | CCAAGCCAACTTTATGTCAGGG | AGCCCGCTTCTTTGTTAATTTGA |
| Ptch1 | AAAGAACTGCGGCAAGTTTTTG | CTTCTCCTATCTTCTGACGGGT |
| Shh | AAAGCTGACCCCTTTAGCCTA | TTCGGAGTTTCTTGTGATCTTCC |
| ChIP‒qPCR | GCTACATGGGCAGAGGACTC | CACCAAGACGACAGCTCCTT |
| Pard3 | GGAGATGGCCGCATGAAAGTT | CTCCAAGCGATGCACCTGTAT |
| Sox2 | GCGGAGTGGAAACTTTTGTCC | CGGGAAGCGTGTACTTATCCTT |
| Oct4 | CACCATCTGTCGCTTCGAGG | AGGGTCTCCGATTTGCATATCT |
| Nanog | TCTTCCTGGTCCCCACAGTTT | GCAAGAATAGTTCTCGGGATGAA |
| Cd133 | CCTTGTGGTTCTTACGTTTGTTG | CGTTGACGACATTCTCAAGCTG |
| Actin | GTTGTCGACGACGAGCG | GCACAGAGCCTCGCCTT |

**Table S2 Patient information** **of tissue microarray cohort**

| Clinicopathological factors |
| --- |
| Age (≥ 60 y, n = 20; < 60 y, n = 70) |
| Sex (male, n = 76; female, n = 16) |
| Tumour size (≥ 3.5 cm, n =64; < 3.5 cm, n = 26) |
| Recurrence (Yes, n=53; no, n=37) |
| HBsAg (positive, n = 71; negative, n = 19) |
| HBcAb (positive, n=80; negative, n=8; N.A, n=2) |
| Cirrhosis (Yes, n=78, no, n=12) |
